# Chemical Proteomics Reveals *N*^ε^-Fatty-Acylation of Septins by Rho Inactivation Domain (RID) of the *Vibrio* MARTX Toxin to Alter Septin Localization and Organization

**DOI:** 10.1016/j.mcpro.2024.100730

**Published:** 2024-02-02

**Authors:** Yaxin Xu, Ke Ding, Tao Peng

**Affiliations:** 1 State Key Laboratory of Chemical Oncogenomics, School of Chemical Biology and Biotechnology, Peking University Shenzhen Graduate School, Shenzhen, China; 2 Shenzhen Bay Laboratory, Institute of Chemical Biology, Shenzhen, China

**Keywords:** chemical proteomics, lysine *N*^ε^-fatty-acylation, Rho inactivation domain (RID), MARTX toxin, septin

## Abstract

*Vibrio* species, the Gram-negative bacterial pathogens causing cholera and sepsis, produce multiple secreted virulence factors for infection and pathogenesis. Among these is the multifunctional-autoprocessing repeats-in-toxin (MARTX) toxin that releases several critical effector domains with distinct functions inside eukaryotic host cells. One such effector domain, the Rho inactivation domain (RID), has been discovered to catalyze long-chain *N*^ε^-fatty-acylation on lysine residues of Rho GTPases, causing inactivation of Rho GTPases and disruption of the host actin cytoskeleton. However, whether RID modifies other host proteins to exert additional functions remains to be determined. Herein, we describe the integration of bioorthogonal chemical labeling and quantitative proteomics to globally profile the target proteins modified by RID in living cells. More than 246 proteins are identified as new RID substrates, including many involved in GTPase regulation, cytoskeletal organization, and cell division. We demonstrate that RID extensively *N*^ε^-fatty-acylates septin proteins, the fourth cytoskeletal component of mammalian cells with important roles in diverse cellular processes. While affinity purification and mass spectrometry analysis show that RID-mediated *N*^ε^-fatty-acylation does not affect septin-septin interactions, this modification increases the membrane association of septins and confers localization to detergent-resistant membrane rafts. As a result, the filamentous assembly and organization of septins are disrupted by RID-mediated *N*^ε^-fatty-acylation, further contributing to cytoskeletal and mitotic defects that phenocopy the effects of septin depletion. Overall, our work greatly expands the substrate scope and function of RID and demonstrates the role of RID-mediated *N*^ε^-fatty-acylation in manipulating septin localization and organization.

## Highlights

• Quantitative chemical proteomics identifies *N*^ε^-fatty-acylation substrates of RID.
• RID targets a broad range of host proteins involved in many cellular processes.
• Septins are *N*^ε^-fatty-acylated on multiple *C*-terminal lysine residues by RID.
• RID-mediated *N*^ε^-fatty-acylation disrupts the organization of septin filaments.
• RID-mediated *N*^ε^-fatty-acylation confers localization of septins to DRMs.

## Graphical Abstract

**Figure:**
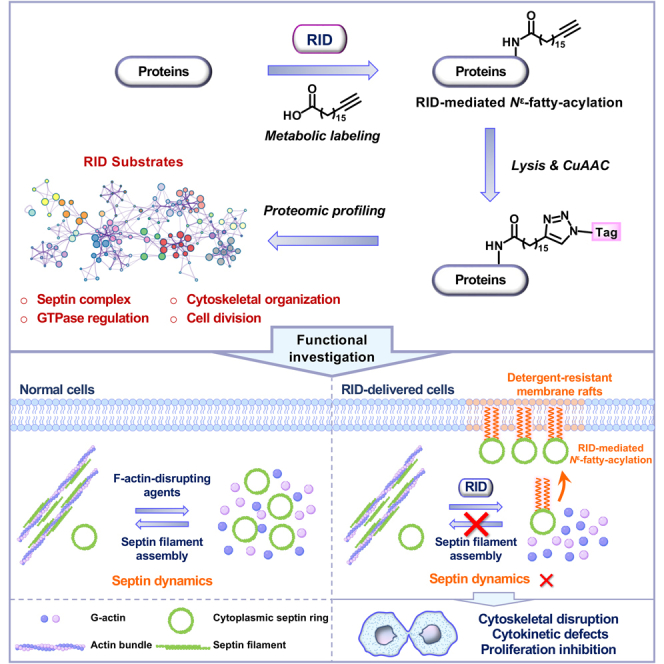

## In Brief

Using bioorthogonal chemical labeling and quantitative proteomic analysis, Xu *et al.* report the identification of septin proteins, the fourth mammalian cytoskeletal component, as the target proteins extensively *N*^ε^-fatty-acylated by Rho Inactivation Domain (RID) of the *Vibrio* MARTX toxin in living cells. Further functional studies demonstrate that RID-mediated *N*^ε^-fatty-acylation confers localization of septins to detergent-resistant membrane rafts and disrupts the filamentous assembly and organization of septins.

The multifunctional-autoprocessing repeats-in-toxin (MARTX) toxins are a class of large toxins that are the critical virulence factors of many Gram-negative bacterial pathogens (1), such as *Vibrio cholerae* and *Vibrio vulnificus* of the *Vibrio* species that cause the diarrheal disease cholera and severe septicemia, respectively (2). RTX toxins are secreted from the bacteria, translocated across the eukaryotic plasma membrane, and autoprocessed by a conserved cysteine protease domain to ultimately release multiple *bona fide* effector domains into host cell cytosol (3, 4, 5). These MARTX toxin effectors exhibit distinct cytopathic and cytotoxic activities that can alter key cellular processes, such as cytoskeletal dynamics (6, 7, 8, 9, 10, 11), guanosine triphosphatase (GTPase) signaling (12, 13), and vesicular trafficking (14), to promote bacterial survival and pathogenesis. Among them is the Rho GTPase inactivation domain (RID), a conserved effector domain in *Vibrio* MARTX toxins, that was found to significantly contribute to virulence (15).

**Fig. 1:**
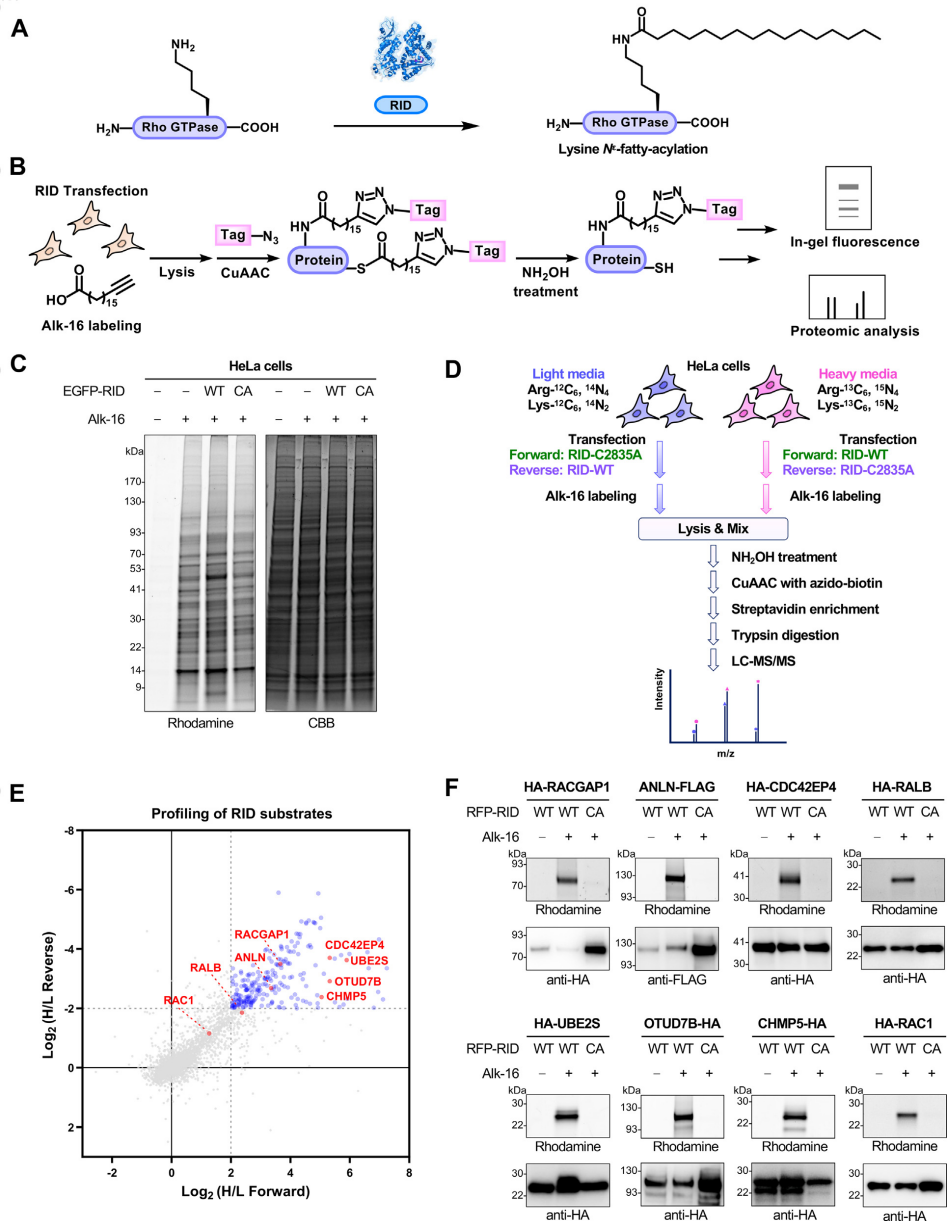
**Chemical proteomics for global profiling of *N***^**ε**^**-fatty-acylation identifies the substrates of RID.***A*, RID of the *Vibrio* MARTX toxin catalyzes *N*^ε^-fatty-acylation modifications on lysines of Rho GTPases. *B*, schematic for in-gel fluorescence and proteomics analyses of RID-mediated *N*^ε^-fatty-acylation of host proteins using metabolic labeling with the bioorthogonal chemical reporter Alk-16 and click reaction. *C*, in-gel fluorescence analysis of Alk-16-labeled fatty-acylated proteins in mock- and RID (WT or CA)-transfected HeLa cells. Coomassie Brilliant blue (CBB) is shown to confirm equal protein loading. *D*, workflow of dual SILAC quantitative chemical proteomics for profiling *N*^ε^-fatty-acylation substrates of RID. In the “Forward” experiment, cells cultured in SILAC heavy and light media were transfected to express RID-WT and RID-CA, respectively. In the “Reverse” experiment, cells cultured in SILAC heavy and light media were transfected to express RID-CA and RID-WT, respectively. *E*, scatter plot of the SILAC quantitative chemical proteomics data. H/L represents the SILAC ratio between heavy and light labels in the indicated Forward or Reverse experiment. Shown in *blue* are protein hits with enrichment of more than four folds, *i.e.*, log_2_ H/L ≥2 in the Forward experiment and log_2_ H/L ≤−2 in the Reverse experiment, in the RID-WT sample relative to the RID-CA sample. Shown in *red* are protein hits validated in this study. *F*, validation of RID-mediated *N*^ε^-fatty-acylation of candidate proteins identified in the quantitative chemical proteomics. Selected candidate proteins were individually co-transfected with RID-WT or RID-CA into HEK293T cells. The cells were metabolically labeled with Alk-16 and subjected to immunoprecipitation and in-gel fluorescence assay. Anti-HA or anti-FLAG immunoblotting is shown to confirm sample loading. Note that the unequal loading was due to the cytotoxicity of RID-WT affecting protein co-expression.

As a dominant effector domain, RID of the *V. cholerae* MARTX toxin was initially discovered to cause cell rounding through inactivation of small Rho GTPases (*e.g.*, Rho, RAC, and Cdc42) and depolymerization of the host actin cytoskeleton (12). Later studies showed that RID consists of two subdomains including a membrane localization subdomain for targeting to plasma membrane and an activity subdomain necessary for cell rounding (16, 17). While bioinformatic analysis predicted folding of the RID activity subdomain as a circularly permuted papain-like thiol protease (18), mutagenesis and biochemical analyses demonstrated that a putative His-Asp-Cys catalytic triad in this subdomain is essential for the function of RID (17). The exact enzymatic activity of RID remained unclear until a seminal work in 2017 discovered that RID of *Vibrios* harbors a lysine *N*^ε^-fatty-acyltransferase activity (19). In particular, it was found that RID from both *V. cholerae* and *V. vulnificus* can efficiently catalyze the covalent attachment of long-chain fatty-acyl groups (*e.g.*, palmitoyl groups) onto the ε-amino position of lysine residues in the *C*-terminal polybasic regions (PBRs) of Rho GTPases (Fig. 1*A*). The resulting modification, referred to as *N*^ε^-fatty-acylation, led to the inactivation of Rho GTPases and disruption of downstream signaling in host cells, which explains the known function of RID in inducing cell rounding (19).

Although first discovered 35 years ago in mammalian cells (20), protein *N*^ε^-fatty-acylation has been understudied in comparison with classical types of long-chain fatty-acylation such as *N*-myristoylation and *S*-palmitoylation that occur on *N*-terminal glycine and cysteine residues (21), respectively. So far, only a handful of *N*^ε^-fatty-acylated proteins have been identified in mammalian cells, namely, secreted cytokines IL-1α (20, 22) and TNF-α (23, 24), aquaporin-0 (25), Ras family proteins (26, 27), and Arf6 (28). Moreover, despite the recent finding that *N*-myristoyltransferases can catalyze *N*^ε^-fatty-acylation of the lysine residue near the *N*-terminus of Arf6 (28), mammalian *N*^ε^-fatty-acyltransferases that broadly act on protein substrates have not been identified yet (29, 30), which, together with the limited substrate scope, has seriously impeded our understanding of this modification.

Interestingly, *N*^ε^-fatty-acyltransferases of bacterial origin have been discovered over the past several years. Such examples include the RID of *Vibrio* species (19) and the *Shigella flexneri* type III secretion system (T3SS) effector IcsB (31). Previously predicted to possess a conserved catalytic triad resembling that in RID (18), IcsB was also found to function as a *N*^ε^-fatty-acyltransferase that utilizes long-chain fatty-acyl groups to modify lysine residues of Rho GTPases (31). Using global and quantitative proteomic analysis, we identified that in addition to Rho GTPases IcsB can modify multiple other host proteins that are involved in a variety of cellular processes. Further knockout and functional characterization indicated that IcsB-mediated *N*^ε^-fatty-acylation of CHMP5 is required for *Shigella* to escape from host autophagy (31). Similar to IcsB, RID was shown to also catalyze *N*^ε^-fatty-acylation of Rho GTPases (19). However, until now Rho GTPases including RAC1–3, RhoA, and Cdc42 are the only known substrates of RID. Whether RID modifies other host proteins to exert additional unknown functions remains to be explored. Considering the crucial role of post-translational modifications (PTMs), especially fatty-acylation (32), manipulated by bacterial effectors in host-pathogen interactions (33, 34), identification of RID substrates should significantly improve our understanding of both RID function and *N*^ε^-fatty-acylation biology.

Herein, we present a chemical proteomics profiling that relies on bioorthogonal chemical labeling and quantitative proteomics to identify host proteins that are specifically *N*^ε^-fatty-acylated by RID in living cells. This global and unbiased analysis reveals a series of unreported RID targets that are involved in various host cellular processes, especially in GTPase regulation, cytoskeletal organization, and cell division. In particular, we show that septins, a family of GTP-binding proteins comprising a unique cytoskeletal and filamentous system, are *N*^ε^-fatty-acylated by RID on lysine residues in their *C*-terminal polybasic regions. RID-mediated *N*^ε^-fatty-acylation increases the membrane affinity of septins, confers localization of septins to detergent-resistant membranes (DRMs), and induces the formation of detergent-resistant septin rings. As a result of these, the organization of septin filaments is disrupted by RID, which further contributes to cytoskeletal collapse and mitotic defects in mammalian cells. Our study thus provides important insights into the targets of RID, the biological function of *N*^ε^-fatty-acylation, and the manipulation of septin organization by RID-mediated *N*^ε^-fatty-acylation.

## Experimental Procedures

### General Reagents, Instruments, and Antibodies

Alk-16, azido-rhodamine, and azido-biotin were synthesized in the lab according to the literature (35). Recombinant DNA cloning was performed with the Seamless Cloning Kit (Beyotime). Oligonucleotide primers and gene fragments were synthesized by Tsingke Biotechnology. Plasmid DNA purification was carried out with the Plasmid Mini Kit (Omega). Ampicillin and kanamycin were purchased from Sangon Biotech. Polyethylenimine (PEI) was purchased from Polysciences. Viafect was purchased from Promega. JetPRIME was purchased from Polyplus. Protease inhibitor cocktail (cOmplete ULTRA mini Tablets, EDTA-free) was purchased from Roche. Benzonase was purchased from HaiGene. For pharmacological treatments, latrunculin B was from Maokangbio. In-gel fluorescence and Western blotting were recorded on a ChemiDoc MP imaging system (Bio-Rad). Fluorescence imaging was performed with a Nikon A1R confocal fluorescence microscope or a Zeiss Elyra 7 microscope. Super-resolution imaging was performed with a Zeiss Elyra 7 with Lattice SIM^2^ super-resolution microscope.

Antibodies used in this study are listed as follows: anti-HA-HRP conjugate (3F10, 1:1000 dilution, Roche); mouse anti-FLAG (F1804, 1:1000 dilution, Sigma); mouse anti-EGFP (66002-1-Ig, 1:10,000 dilution, Proteintech); rabbit anti-mCherry (26765-1-AP, 1:10,000 dilution, Proteintech); anti-GAPDH-HRP (HRP-60004, 1:10,000 dilution, Proteintech); rabbit anti-calnexin (10427-2-AP, 1:1000 dilution, Proteintech); rabbit anti-H3 (17168-1-AP, 1:2000 dilution, Proteintech); rabbit anti-flotillin1 (15571-1-AP, 1:1000 dilution, Proteintech); mouse anti-SEPT2 (PTM-5434, 1:5000 dilution, PTM Biolabs); rabbit anti-SEPT6 (12805-1-AP, 1:1000 dilution, Proteintech); rabbit anti-SEPT7 (13818-1-AP, 1:1000 dilution, Proteintech); rabbit anti-SEPT9 (10769-1-AP, 1:2000 dilution for Western blotting, 1:500 dilution for immunofluorescence, Proteintech); donkey anti-rabbit-IgG-HRP secondary antibody (711-035-152, 1:10,000 dilution, Jackson ImmunoResearch); donkey anti-mouse-IgG-HRP secondary antibody (735-035-150, 1:10,000 dilution, Jackson ImmunoResearch); rabbit anti-HA (3724, 1:800 dilution, Cell Signaling Technology); mouse anti-tubulin (AT819, 1:1000 dilution, Beyotime); Alexa Fluor 555-conjugated goat anti-rabbit secondary antibody (A32732, 1:1000 dilution, Invitrogen); Alexa Fluor 555-conjugated goat anti-mouse secondary antibody (A32727, 1:1000 dilution, Invitrogen).

### Plasmid and Cloning

Full-length RIDvv DNA (residues 2283–2898 from the *V. vulnificus* MARTX toxin) was kindly provided by Professor Yongqun Zhu at Zhejiang University (19). IcsB DNA was kindly provided by Professor Feng Shao at the National Institute of Biological Science (31). RIDvv and IcsB were cloned into either a modified pEGFP-C1 vector containing a FLAG tag preceding EGFP or the pCS2 vector with an *N*-terminal RFP tag. The mCherry-actin plasmid was a gift from Michael Davidson and obtained from Addgene (plasmid #54967).

DNA fragments for plasmid construction were purchased from MiaoLing Plasmid Platform unless otherwise noted. ANLN was cloned into the pEnCMV vector with a *C*-terminal 3×FLAG tag. CHMP5 and OTUD7B were cloned into the pCMV-HA-C vector. UBE2S, RALB, CDC42EP4, and RACGAP1 were cloned into the pCMV-HA-N vector. SEPT6, SEPT7, SEPT8, SEPT9, and SEPT11 were cloned into the pEmerald-N1 vector, while SEPT2 and SEPT10 were cloned into the pCMV vector with an *N*-terminal EGFP tag. For immunofluorescence, SEPT2, SEPT6, and SEPT7 were cloned into the pCMV-HA-C vector. Site-directed mutagenesis was performed with the Q5 Site-Directed Mutagenesis Kit (New England Biolabs) using primers designed by NEBaseChanger. Oligonucleotide primers were synthesized by Tsingke Biotechnology, and all plasmids were verified by DNA sequencing.

### Cell Culture and Transfection

HEK293T and HeLa cells were purchased from ATCC and grown in DMEM (Dulbecco’s modified Eagle’s medium; Corning, cat#10-013-CVR) supplemented with 10% FBS (fetal bovine serum; Corning, cat#35-076-CV) at 37 °C with an atmosphere of 5% CO_2_ in a humidified incubator. For transfection, HEK293T cells were grown in cell culture dishes or plates to about 70% confluence and transfected with indicated plasmids using PEI (Polysciences) in Opti-MEM media (ThermoFisher) at a 2.5:1 ratio of PEI/DNA for about 18 to 24 h. HeLa cells were transfected with a 2.5:1 ratio of transfection reagent/DNA using Viafect (Promega) or jetPRIME (Polyplus) according to the manufacturer’s protocol in Opti-MEM media or jetPRIME buffer at 70% confluence.

### Click Reaction and In-Gel Fluorescence Analysis

HeLa cells were transfected with the plasmids of interest using Viafect (Promega, 2.5 μl per well) in 12-well plates (Corning). After 6 h of incubation, the cells were labeled with 50 μM Alk-16 in DMEM supplemented with 2% charcoal/dextran treated FBS (ThermoFisher) for 16 h at 37 °C. Following this, the cells were harvested, washed with cold PBS, and lysed in 1% SDS buffer (1% SDS, 150 mM NaCl, 50 mM HEPES, pH 7.4, supplemented with benzonase) with vigorous vortexing. The resulting lysates were centrifuged at 12,000*g* for 20 min at room temperature to remove cellular debris and the protein concentrations were determined by the BCA assay (Pierce). To perform the click reaction, 100 μg of cell lysates were mixed with 1% SDS buffer (1% SDS, 150 mM NaCl, 50 mM HEPES, pH 7.4) to 50 μl, and 39 μl of HEPES buffer (150 mM NaCl, 50 mM HEPES, pH 7.4) was added to make a final volume of 89 μl. The lysates were then combined with a freshly prepared click reaction cocktail containing azido-rhodamine (2 μl, 10 mM stock solution in DMSO), tris-(2-carboxyethyl) phosphine hydrochloride (TCEP, 2 μl, 50 mM freshly prepared stock solution in ddH_2_O), tris-[(1-benzyl-1H-1,2,3-triazol-4-yl)methyl]amine (TBTA, 5 μl, 10 mM stock solution in DMSO/t-butanol), and CuSO_4_·5H_2_O (2 μl, 50 mM freshly prepared stock solution in ddH_2_O). The reaction mixture was incubated in the dark at room temperature for 2 h. The click reaction was terminated by adding 500 μl of ice-cold methanol and storing it at −20 °C overnight. The protein mixture was centrifuged at 20,000*g* for 20 min at 4 °C to pellet the proteins, and the supernatants were discarded. The protein pellets were washed twice with ice-cold methanol and air-dried. The resulting protein pellets were resuspended in 57.5 μl of 4% SDS lysis buffer (4% SDS, 150 mM NaCl, 50 mM triethanolamine, pH 7.4) and 12.5 μl of NH_2_OH (2 M), and then diluted with 25 μl of 4× SDS-loading buffer (40% glycerol, 200 mM Tris-HCl, pH 6.8, 8% SDS, 0.4% bromophenol blue) and 5 μl of 2-mercaptoethanol. The resulting samples were heated for 5 min at 95 °C before loading onto 4 to 20% Bis-Tris gels (Genscript) for SDS-PAGE separation. Generally, 20 μg of protein per gel lane was loaded for in-gel fluorescence visualization. The gels were scanned on a ChemiDoc MP Imager (Bio-Rad) using the rhodamine filter. Following in-gel fluorescence scanning, the gels were stained with Coomassie Brilliant Blue staining reagent.

### Chemical Proteomic Profiling of RID Substrate Proteins

HeLa cells were grown in DMEM supplemented with 10% FBS at 37 °C in a humidified incubator with an atmosphere of 5% CO_2_. For SILAC experiments, arginine- and lysine-deficient DMEM (ThermoFisher) supplemented with 10% dialyzed FBS (ThermoFisher) was used. For the culture of “light”-labeled cells, SILAC medium containing ^12^C_6_-L-lysine-2HCl (Lys0; 0.80 mM; Sigma) and ^12^C_6_-L-arginine-HCl (Arg0; 0.40 mM; Sigma) was used; for the culture of “heavy”-labeled cells, SILAC medium containing ^13^C_6_,^15^N_2_-L-lysine-2HCl (Lys8; 0.80 mM; Cambridge Isotope) and ^13^C_6_,^15^N_4_-L-arginine-HCl (Arg10; 0.40 mM; Cambridge Isotope) was used. After seven cell doublings, the incorporation of heavy isotopes was estimated to be greater than 98% using liquid chromatography MS/MS (LC-MS/MS) analysis.

To conduct SILAC proteomics studies, “heavy”- and “light”-labeled cells were transfected with Flag-tagged RID-WT and RID-C2835A mutant, respectively, in the “forward” experiment; “heavy”- and “light”-labeled cells were transfected with FLAG-tagged RID-C2835A mutant and RID-WT, respectively, in the “reverse” experiment. Subsequently, the cells were treated with Alk-16 (50 μM) for 16 h. The cells were harvested and lysed with 4% SDS (150 mM NaCl, 50 mM triethanolamine, pH 7.4) through rigorous vortexing and sonication. The cell lysates were then centrifuged at 16,000*g* for 20 min at room temperature to remove cellular debris and protein concentrations were determined using the BCA assay (Pierce). After determining the protein concentration, a mixture of “heavy”-labeled (5.2 mg) and “light”-labeled cell lysates (5.2 mg) was treated with freshly prepared NH_2_OH (0.75 M) at room temperature with rotation for 1 h. Subsequently, the proteins were precipitated using ice-cold methanol overnight, washed six times with ice-cold methanol, air-dried, resuspended in 1% SDS (1% SDS, 150 mM NaCl, 50 mM HEPES, pH 7.4), and clicked with azido-biotin using CuAAC reactions as described above. The proteins were again precipitated with ice-cold methanol. The methanol-washed protein pellets were subsequently resuspended in 4% SDS buffer. Finally, equal amounts of proteins were diluted 1/4 by volume with 50 mM triethanolamine buffer to make a final SDS concentration of 1%. 200 μl of prewashed streptavidin agarose beads (ThermoFisher) were added to each sample, and incubated for 4 h at room temperature with end-over-end rotation. The beads were then washed five times with 1% SDS (in PBS, pH 8.0) with rotation for 5 min and transferred into spin-columns (ThermoFisher). The beads were then washed with 5 M urea (50 mM Tris-HCl, pH 8.0), followed by washes with PBS (pH 7.4) and 100 mM ammonium bicarbonate (ABC) buffer. The beads were then transferred into new 1.5 ml centrifuge tubes and treated with 10 mM DTT for 1.5 h, followed by 20 mM iodoacetamide treatment for another 1.5 h in the dark. Afterward, the beads were washed with 100 mM ABC buffer and digested with 130 ng of trypsin (Promega) in ABC buffer (100 μl) at 37 °C overnight. The resulting supernatants were collected, dried, and subjected to fractionation with SCX StageTips for LC-MS/MS analysis.

The LC-MS/MS analysis was conducted using an EASY-nLC 1200 system (ThermoFisher) coupled to a Q Exactive HF-X Hybrid Quadrupole-Orbitrap mass spectrometer (ThermoFisher). Peptide samples were loaded onto an Acclaim PepMap RSLC C18 reverse-phase column (75 μm × 25 cm, nanoViper, C18, 2 μm, 100 Å; ThermoFisher). HPLC separation was performed with a 120-min gradient, starting with 90% buffer A (water with 0.1% formic acid) and 10% buffer B (80% acetonitrile in water with 0.1% formic acid). The gradient increased to 45% buffer B over 30 min, followed by a further increase to 95% buffer B over the next 70 min. The system was maintained at 95% buffer B for 20 min. The samples were injected into the mass spectrometer at a flow rate of 0.3 μl/min.

The mass spectrometer calibration was performed using the Tune instrument control software. The spray voltage was set to 2.1 kV, the heated capillary temperature was maintained at 320 °C, and the funnel RF level was set at 40. The mass spectrometer was configured for data-dependent acquisition mode using the full MS/DD–MS/MS setup. All data were acquired in profile mode with positive polarity. The full MS resolution was set to 60,000 at m/z 200, and the full MS automatic gain control (AGC) target was set to 3 × 10^6^ with a maximum ion injection time (IT) of 50 ms. The mass range for data acquisition was set from 350 to 1800 m/z. The MS2 resolution was set to 15,000 at m/z 200, the AGC target value for MS2 was set to 1 × 10^5^, and the intensity threshold at 2.2 × 10^4^. The isolation width was set at 1.6 m/z, and a fixed first mass of 100 m/z was used. The normalized collision energy was set at 28%. Peptide match was set to preferred, and isotope exclusion was enabled. Precursor ions with unassigned, single, or six and higher charge states were excluded from fragmentation selection. The dynamic exclusion time was set to 30 s.

Acquired MS raw files were analyzed by MaxQuant software v1.5.3.8 (36) using the Andromeda search engine and searched against the Human UniProt Reference Proteome database (UP000005640, modified on January 5, 2021; 20,609 entries, one protein sequence per gene without isoforms) concatenated with common known contaminants. Multiplicity was set to 2 with heavy labels of Arg10 and Lys8. Enzyme specificity was defined as trypsin with a maximum allowance of two missed cleavages. Carbamidomethylation of cysteine was selected as a fixed modification, while methionine oxidation and *N*-terminal acetylation were set as variable modifications. The first search peptide tolerance was set at 20 ppm, and the main search peptide tolerance was set at 4.5 ppm. The allowed fragment mass deviation was 20 ppm. The minimum length of peptides was set to seven amino acids, and the maximum mass was set to 4600 Da. False discovery rates (FDRs) were controlled at 1% for peptide spectrum match, protein, and site decoy fraction levels. Only unique and razor peptides were used for quantification with minimal two-ratio counts. The “Re-quantify” feature of MaxQuant was utilized to correct the quantification of proteins. All other parameters in MaxQuant were maintained at their default values. The search results obtained from MaxQuant were further processed using Perseus v1.6.10.0 (http://www.perseus-framework.org/). In this step, known contaminants, reverse hits, and hits identified only by site were excluded from the analysis. Proteins identified with only one unique peptide were also excluded from the analysis. SILAC ratios were subjected to log2 transformation.

### Western Blot

Cells were generally lysed with 4% SDS lysis buffer containing a protease inhibitor cocktail and benzonase unless otherwise stated. Cell lysates were clarified by centrifugation at 12,000*g* for 20 min and quantified by the BCA assay (Pierce). Equal amounts of cell lysates were diluted with 4× reducing SDS-loading buffer and β-mercaptoethanol. The samples were heated for 10 min at 95 °C and loaded onto 4 to 20% Bis-Tris gels (Genscript) for SDS-PAGE separation. Gels were transferred onto nitrocellulose membranes using the Bio-Rad Trans-Blot Turbo Transfer System (25 V, 30 min). After blocking with 5% nonfat milk in PBST (0.05% Tween-20 in PBS) for 30 min at room temperature, the membranes were incubated overnight with primary antibodies at 4 °C. Following PBST washes three times, appropriate secondary antibodies were added, and the membranes were developed with ECL reagents (Yeasen). Membranes were imaged using a ChemiDoc MP Imager (Bio-Rad). The protein band of interest was quantified using ImageLab software (Bio-Rad).

### Immunoprecipitation and In-Gel Fluorescence Analysis

HEK293T cells grown at 70% confluence in 60 mm dishes were co-transfected with RID substrates and RID-WT or RID-C2835A. After 6 h incubation, cells were labeled with 50 μM Alk-16 in DMEM supplemented with 2% charcoal/dextran treated FBS (ThermoFisher) for 16 h at 37 °C. The HEK293T cells were collected and rinsed with chilled PBS. The cells were lysed with RIPA buffer (1% Triton X-100, 1% sodium deoxycholate, 0.1% SDS, 150 mM NaCl, 50 mM triethanolamine, pH 7.4) supplemented with protease inhibitor cocktail (Roche) and benzonase with vigorous vortexing. After centrifugation at 12,000*g* for 20 min at 4 °C to remove cellular debris, protein concentrations were quantified using the BCA assay (Pierce). Anti-HA agarose beads (ThermoFisher), anti-EGFP nanobody agarose beads (AlpalifeBio), and anti-FLAG agarose beads (Sigma) were used to immunoprecipitate HA-tagged, EGFP-tagged, and FLAG-tagged proteins, respectively, from approximately 600 μg of cell lysates. The beads were incubated on a rotator at 4 °C for 2 h and then washed three times with 1 ml of chilled RIPA buffer, followed by three additional washes with PBS. The beads were resuspended with 44.5 μl of PBS buffer and incubated with 5.5 μl of freshly prepared click reaction cocktail as described above in the dark for 2 h at room temperature. Following the reaction, the beads were washed three times with 1 ml of RIPA buffer, resuspended with 17.25 μl of 4% SDS buffer (4% SDS, 150 mM NaCl, 50 mM triethanolamine, pH 7.4), and diluted with 7.5 μl of 4× SDS-loading buffer, 1.5 μl of 2-mercaptoethanol, and 3.75 μl of NH_2_OH (2 M). The mixture was heated for 5 min at 95 °C. 20 μl of the supernatant was loaded per gel lane for separation by SDS-PAGE and in-gel fluorescence scanning. The rest was loaded onto another gel for Western blotting analysis. For analysis of the turnover of SEPT7 *N*^ε^-fatty-acylation, HEK293T cells were co-transfected with RFP-RID and SEPT7-EGFP. After 10 h incubation, cells were labeled with 100 μM Alk-16 in DMEM supplemented with 2% charcoal/dextran treated FBS (ThermoFisher) for 4 h at 37 °C. After the removal of Alk-16 labeling media, DMEM containing 10% FBS and 100 μM palmitic acid (Sigma) was added. The cells were harvested at the indicated time points. Immunoprecipitation and in-gel fluorescence analysis were performed as described above.

### Bioinformatics Analysis

DAVID bioinformatics resources (https://david-d.ncifcrf.gov/, DAVID 2021, Knowledgebase v2023q4) were used to perform GO term, KEGG pathway, and PTM enrichment analyses (37). Protein class analysis was carried out by PANTHER version 18.0 (http://www.pantherdb.org/, 2023 update) (38). Functional enrichment analysis and protein interaction network analysis were performed by Metascape version 3.5 (https://metascape.org, 2024 update) (39).

### Subcellular Fractionation

Subcellular fractionation by differential detergent lysis was performed as described previously (40). Specifically, HEK293T cells were cultured overnight in 60 mm dishes and transfected with indicated plasmids for 24 h. Cells were harvested, washed with cold PBS twice, and pelleted by centrifugation at 4000*g* for 2 min. To obtain the cytosolic fraction, cell pellets were permeabilized with 100 μl of ice-cold digitonin extraction buffer (5 mM digitonin, 5 mM EDTA, 10 mM PIPES, pH 6.8, 1 mM PMSF) and centrifuged at 400*g* and 4 °C for 10 min. The resulting supernatant was collected and stored at 4 °C. The pellets were washed with 200 μl of ice-cold digitonin extraction buffer and centrifuged at 4000*g* for 2 min. The supernatant was discarded to leave the digitonin-insoluble pellets. To obtain the membrane fraction, the digitonin-insoluble pellets were resuspended in 60 μl of ice-cold Triton X-100 extraction buffer (0.5% Triton X-100, 3 mM EDTA, 10 mM PIPES, pH 7.4, 1 mM PMSF) and incubated with gentle agitation for 30 min. The mixture was centrifuged at 5000*g* and 4 °C for 10 min, and the supernatant was collected and stored at 4 °C. The pellets were washed with 200 μl of ice-cold Triton X-100 extraction buffer and centrifuged at 10,000*g* for 1 min. The supernatant was discarded to leave the Triton-insoluble pellets, which were finally dissolved in 60 μl of 4% SDS lysis buffer (4% SDS, 150 mM NaCl, 50 mM TEA, pH 7.4). The protein concentration of each fraction was determined using the BCA assay (Pierce) and normalized to a concentration of 0.8 μg/μl. Samples were heated for 5 min at 95 °C and loaded onto 4 to 20% Bis-Tris gels (Genscript) for SDS-PAGE separation and Western blot analysis.

### Protein Flotation Assay

Separation of DRMs by density centrifugation in OptiPrep was performed as described previously (41). HEK293T cells were cultured overnight in 100 mm dishes and transfected with indicated plasmids for 16 h. Cells were harvested, washed with cold PBS, and pelleted by centrifugation at 4000*g* for 2 min. The cell pellets were resuspended in 500 μl TNE buffer (150 mM NaCl, 2 mM EDTA, 50 mM Tris-HCl, pH 7.4) supplemented with a protease inhibitor cocktail and homogenized with a 25-G needle for 25 strokes. The cell homogenate was transferred to a new microfuge tube and 60 μl of 10% Triton X-100 was added. The mixture was gently mixed and further incubated for 30 min. The sample was then diluted with 1 ml of OptiPrep (60% iodixanol) (Serumwerk Bernburg AG). 900 μl of the mixture was transferred to an MLS-50 centrifuge tube (Beckman), overlaid with 1.8 ml of 30% iodixanol and 300 μl of TNE buffer, and centrifuged at 259,000*g* with an MLS-50 rotor (Beckman) for 2 h. Three 1-mL fractions were collected from the top to the bottom. The uppermost fraction contained the DRMs, while the lowermost fraction contained detergent-soluble proteins. After centrifugation at 12,000*g* for 20 min at 4 °C and concentration quantification using the BCA assay (Pierce), the fractions were normalized to the same concentration and separated by SDS-PAGE for Western blot analysis.

### Co-immunoprecipitation

Cells were harvested after transfection for 20 h, washed with PBS twice, and lysed on ice for 30 min with Triton X-100 lysis buffer (1% Triton X-100, 150 mM NaCl, 50 mM Tris-HCl, pH 7.4) supplemented with protease inhibitor cocktail. The cell lysates were centrifuged at 12,000*g* for 20 min at 4 °C and the cleared cell lysates were quantified using the BCA assay (Pierce). Equal amounts of cell lysates were incubated with anti-EGFP nanobody conjugated agarose beads (AlpalifeBio) at 4 °C for 2 h with end-over-end rotation. After washing with Triton X-100 lysis buffer five times, the beads were eluted with 1× reducing SDS-loading buffer for SDS-PAGE separation and Western blot analysis.

### Affinity Purification-Mass Spectrometry (AP-MS) Analysis

For AP-MS analysis of SEPT6, HEK293T cells were cultured in 150 mm dishes to ∼80% confluency, and transfected with indicated plasmids for 20 h. Cells were harvested and affinity purification was performed as described above for co-immunoprecipitation. The resulting samples were loaded onto 4 to 20% Bis-Tris gels (Genscript). Electrophoresis was carried out at a constant voltage of 140 V for 8 min. The proteins were visualized with Coomassie blue staining (Beyotime). The gel lanes were diced into small pieces (*ca.* 1 × 1 mm), distained with 80 mM ammonium bicarbonate buffer (ABC) in 40% acetonitrile, dehydrated with 100% acetonitrile for 10 min, dried under vacuum centrifugation, and subjected to in-gel reduction and alkylation with 20 mM DTT for 30 min and 50 mM iodoacetamide for another 30 min. After washing with 100 mM ABC, gel pieces were dehydrated again and dried as described above. Afterward, the gel slices were digested with 130 ng of trypsin (Promega) in 100 mM ABC (100 μl) at 37 °C overnight. The tryptic peptides were extracted with 40% acetonitrile containing 4% formic acid and dried under vacuum centrifugation for LC-MS/MS analysis (42).

The LC-MS/MS analysis was conducted using an EASY-nLC 1200 system (ThermoFisher) coupled to a Q Exactive HF-X Hybrid Quadrupole-Orbitrap mass spectrometer (ThermoFisher). The LC conditions and mass spectrometer settings are described as above.

Acquired MS raw files were analyzed by MaxQuant software v1.5.3.8 (36) and searched against the Human UniProt Reference Proteome database (UP000005640, modified on January 5, 2021; 20,609 entries, one protein sequence per gene without isoforms) concatenated with common known contaminants. Enzyme specificity was defined as trypsin with a maximum allowance of two missed cleavages. Carbamidomethylation of cysteine was selected as a fixed modification, while methionine oxidation and *N*-terminal acetylation were set as variable modifications. The first search peptide tolerance was set at 20 ppm, and the main search peptide tolerance was set at 4.5 ppm. The allowed fragment mass deviation was 20 ppm. The minimum length of peptides was set to seven amino acids, and the maximum mass was set to 4600 Da. False discovery rates (FDRs) were controlled at 1% for peptide spectrum match, protein, and site decoy fraction levels. Only unique and razor peptides were used for quantification with minimal two-ratio counts. All other parameters in MaxQuant were maintained at their default values. Label-free quantification (LFQ) was performed with the label-free MaxLFQ algorithm in the MaxQuant software as described (43). The search results were analyzed by Perseus v1.6.10.0. Known contaminants, reverse hits, and hits identified only by site were excluded from the analysis. Proteins identified with only one unique peptide were also excluded from the analysis. Logarithmized LFQ intensities were used for measuring protein abundance. Missing values were imputed with random numbers from a normal distribution. Significantly enriched proteins were determined by a volcano plot-based strategy, which combined *t-*test *p* values with folds of enrichment.

### Immunofluorescence

HeLa cells grown on glass coverslips in 12-well plates were transfected with the indicated plasmids using Viafect (Promega) for 16 h. In the case of latrunculin B treatment, the HeLa cells were treated with 1 μM latrunculin B for 30 min before being washed with PBS (44). Cells were then washed with PBS three times, and fixed with 4% paraformaldehyde (ThermoFisher) for 10 min at room temperature. For Triton X-100 treatment, the cells were treated with cold PBS containing 0.1% Triton X-100 for 1 min and then fixed with 4% paraformaldehyde (45). Cells were washed with PBS, permeabilized with 0.1% Triton X-100, and blocked with PBS containing 5% BSA and 0.1% Tween-20. Cells were incubated with primary antibodies overnight at 4 °C, washed three times with 0.1% PBST (PBS containing 0.1% Tween-20), and incubated for 1 h at room temperature with Alexa Fluor 555-conjugated anti-mouse or anti-rabbit secondary antibodies. F-actin was stained with Alexa Fluor 488-phalloidin (YP0059S, 1:200 dilution, Uelandy) for 30 min at room temperature. All antibodies were diluted in the blocking buffer. The samples were then washed with 0.1% PBST three times and were mounted with Antifade Mounting Medium with DAPI (Beyotime). For confocal imaging, the samples were analyzed on a Nikon A1R confocal microscope using a 100× objective; for super-resolution imaging, the samples were analyzed with a Zeiss Elyra 7 with Lattice SIM^2^ super-resolution microscope using a 63× objective. Images were processed using Nikon NIS-Elements and Zeiss ZEN Blue software.

### Cell Proliferation Assay

The HEK293T cells were transfected with the indicated plasmids in 35 mm dishes for 16 h. Afterward, the cells were detached using trypsin solution at room temperature for 2 min and counted to prepare the cell suspension. Subsequently, cells were seeded into 96-well plates at 3000 cells/well using 100 μl of the cell suspension. The plates were then incubated in a humidified incubator with 5% CO_2_ at 37 °C until the cells adhered to the bottom of the wells. Cell proliferation was measured every 24 h using the CCK-8 assay (MCE). For each well, 10 μl of CCK-8 reagent was added and incubated with the cells at 37 °C for 2 h. The absorbance of each well was measured at 450 nm using a microplate reader.

### Experimental Design and Statistical Rationale

Samples for SILAC proteomics represent one biological replicate analyzed in Forward and Reverse experiments. Samples for LFQ proteomics represent three independent biological replicates. All data were shown as mean ± standard deviation, calculated from biological replicates. GraphPad Prism was used for statistical analysis. The methods used for determining error bars and significance were indicated in the corresponding figure legends, with the biological replication number specified. Significance was denoted as follows: *ns* = non-significant (*p* > 0.05), ∗*p* < 0.05, ∗∗*p* < 0.01, ∗∗∗*p* < 0.001, and ∗∗∗∗*p* < 0.0001.

## Results

### Quantitative Chemical Proteomics Identifies the *N*^ε^-Fatty-Acylation Substrates of RID

To globally profile the *N*^ε^-fatty-acylation substrate proteins of RID, we utilized the bioorthogonal chemical reporter Alk-16 (Fig. 1*B*), an alkynyl-functionalized fatty acid analog, to metabolically label fatty-acylated proteins in living cells (35, 46, 47). As a classical method for intracellular delivery of RID (12, 17), cells were transfected to express wild-type (WT) RID of the *V. vulnificus* MARTX toxin or its inactive mutant RID-C2835A (RID-CA) (supplemental Fig. S1, *A* and *B*). The cells were then incubated with Alk-16 overnight and lysed. To minimize the interference from *S*-palmitoylated proteins, the lysates were treated with hydroxylamine (NH_2_OH) which can selectively cleave thioester bonds. The proteins in cell lysates were then subjected to Cu(I)-catalyzed alkyne-azide cycloaddition (CuAAC) reaction (48), *i.e.*, click reaction, with azido-rhodamine or azido-biotin for in-gel fluorescence or proteomics analysis, respectively (Fig. 1*B*). The in-gel fluorescence analysis showed that many proteins were specifically labeled by Alk-16 only in the lysates of cells expressing RID-WT but not in the lysates from RID-CA-expressing cells (Fig. 1*C*), suggesting that RID mediates *N*^ε^-fatty-acylation of these host proteins in living cells.

To identify the substrate proteins of RID, we sought to perform quantitative chemical proteomics experiments by combining the Alk-16 metabolic labeling and stable isotope labeling by amino acids in cell culture (SILAC) (31). To eliminate potential false positives due to isotope labeling and to increase identification reliability, we designed a dual SILAC workflow (Fig. 1*D*). In the “Forward” SILAC experiment, cells were cultured in standard SILAC heavy and light media before transfection to express RID-WT and RID-CA, respectively. In the “Reverse” SILAC experiment, the isotope labeling was switched and the heavy- and light-labeled cells were transfected with RID-CA and RID-WT, respectively. Cells were labeled with Alk-16 and the lysates were then mixed equally. The combined lysates were treated with NH_2_OH, followed by click reaction with azido-biotin, streptavidin enrichment, and trypsin digestion. The resulting peptide samples were analyzed by mass spectrometry for protein identification. The heavy-to-light (H/L) SILAC ratios of identified proteins were quantified to evaluate the extent of enrichment (Fig. 1*D*). Notably, these dual SILAC proteomics analyses identified and quantified a total of 246 host proteins that were repeatedly and significantly enriched (*i.e.*, log_2_ H/L ≥2 and log_2_ H/L ≤−2 in Forward and Reverse experiments, respectively) in a RID-dependent manner and thus represent potential *N*^ε^-fatty-acylation substrates of RID (Fig. 1*E* and supplemental Table S1).

We chose several highly enriched proteins and performed in-gel fluorescence assays to validate the proteomics data. For this, cells were transfected to co-express RID and HA- or FLAG-tagged candidate proteins and labeled with Alk-16. Cell lysates were subjected to immunoprecipitation, reacted with azido-rhodamine, and analyzed by in-gel fluorescence after NH_2_OH treatment (supplemental Fig. S1*C*). The results showed that seven selected candidates, including RACGAP1, ANLN, CDC42EP4, RALB, UBE2S, OTUD7B, and CHMP5 (Fig. 1*E*), were all labeled by Alk-16 only in the presence of RID-WT, but not upon expression of RID-CA (Fig. 1*F*), thus confirming the reliability of our quantitative chemical proteomics for identifying RID substrates. The previously reported RID substrate, that is, RAC1 (19), a Rho family small GTPase, was also retrieved in the proteomics data with modest SILAC ratios (*i.e.*, log_2_ H/L = 1.26 and log_2_ H/L = −1.16 in Forward and Reverse experiments, respectively) (supplemental Table S1). Consistent with this, the in-gel fluorescence analysis confirmed *N*^ε^-fatty-acylation of RAC1 by RID (Fig. 1*F*). Moreover, RAC1 was less modified than ANLN and OTUD7B which were identified with high SILAC ratios (supplemental Fig. S1*D*). Overall, our quantitative chemical proteomics reveals that RID modifies a large number of host proteins in living cells in addition to the known RID substrates, *i.e.*, Rho GTPases.

### Bioinformatics Analysis Indicates that RID Targets a Broad Range of Host Proteins Involved in Many Cellular Processes

**Fig. 2:**
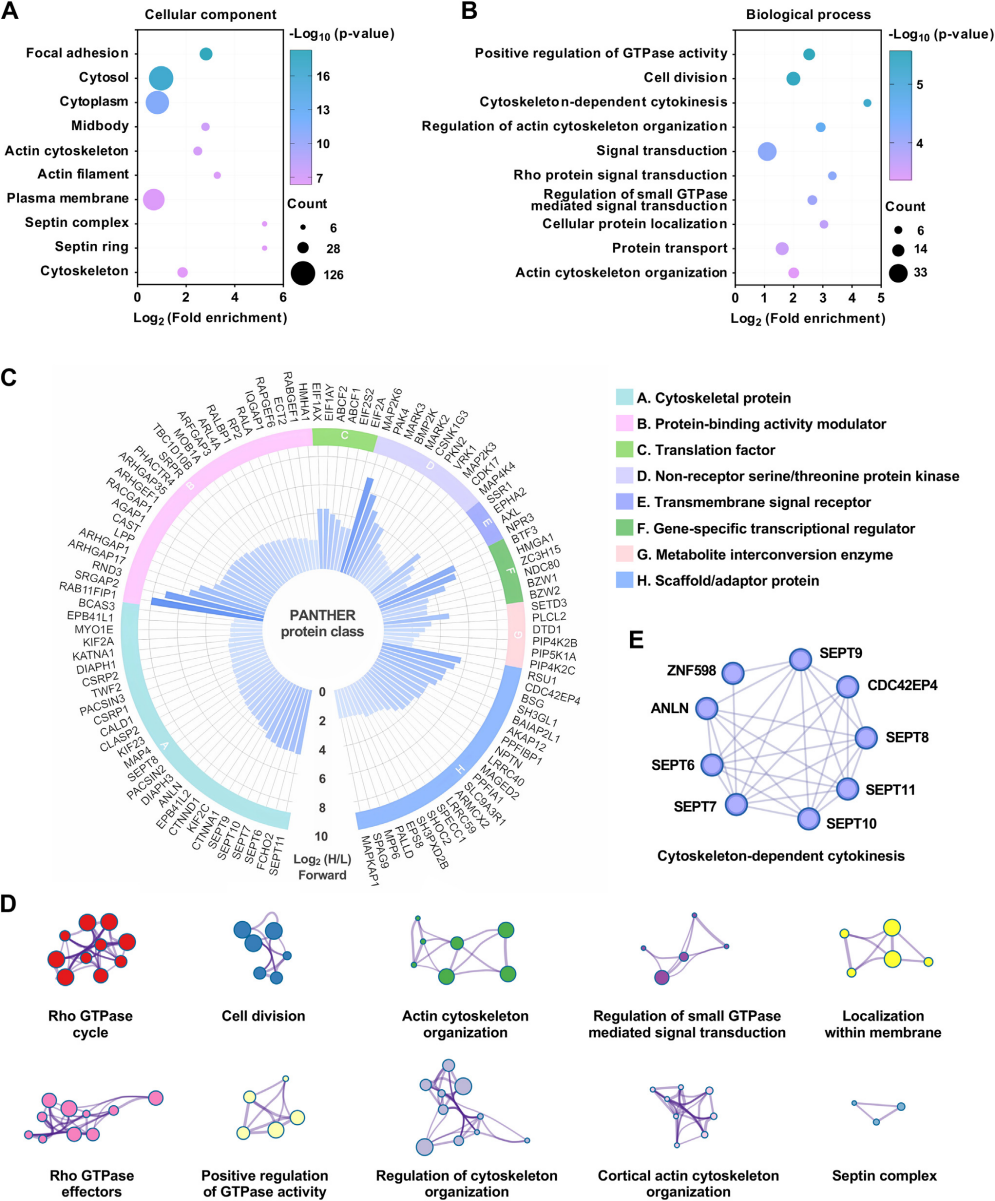
**Bioinformatics analysis of the candidate RID substrates indicates that RID targets a broad range of proteins involved in various host cellular processes.***A*, enrichment analysis of the candidate RID substrates on GO Cellular Component terms. *B*, enrichment analysis of the candidate RID substrates on GO Biological Process terms. *C*, PANTHER protein class enrichment analysis of the candidate RID substrates. *D*, network of the candidate RID substrates shown as clustered functional categories. Each node represents an ontology term enriched in the candidate RID substrates as analyzed by Metascape and nodes are connected by similarities. Size of node represents the extent of enrichment. *E*, the septin protein complex identified in the network of the candidate RID substrates by Metascape. Significantly enriched ontology terms are combined to annotate the putative biological role of the septin protein complex.

To understand the newly identified protein substrates of RID, we performed a series of bioinformatics analyses on these proteins. Gene Ontology (GO) (49) analysis demonstrated that RID substrates are broadly localized in focal adhesion, cytosol, cytoskeleton, midbody, and membrane (Fig. 2*A* and supplemental Table S2*A*) and are mainly involved in biological processes such as cell division, regulation of GTPase activity, cytokinesis, signal transduction, actin cytoskeleton organization, and actin filament bundle assembly (Fig. 2*B* and supplemental Table S2*A*). In addition, GO analysis on molecular functions suggested a significant enrichment in functions like cadherin, protein, RNA, actin, and GTPase binding (supplemental Fig. S2*A* and supplemental Table S2*A*). Moreover, the Kyoto Encyclopedia of Genes and Genomes (KEGG) (50) analysis also indicated that RID substrates are largely associated with pathways such as bacterial invasion/infection, shigellosis, endocytosis, adherens junction, and regulation of actin cytoskeleton (supplemental Fig. S2*B* and supplemental Table S2*A*).

We also carried out a protein class enrichment analysis (38) to describe the classification of RID substrate proteins. Notably, cytoskeletal proteins, protein-binding activity modulators, translation factors, and scaffold/adaptor proteins are highly represented (Fig. 2*C* and supplemental Table S2*B*). Next, we used Metascape (39) to perform the functional enrichment analysis on the candidate RID substrates. A set of top enriched non-redundant clusters of GO biological process terms and KEGG pathways were obtained, and the enrichment networks of the top 20 clusters were created (supplemental Fig. S2*C* and supplemental Table S2*C*), of which the top five most significantly enriched functional clusters are Rho GTPase cycle, cell division, actin cytoskeleton organization, regulation of small GTPase-mediated signal transduction, and localization within membrane (Fig. 2*D* and supplemental Fig. S2*C*). Other notable enriched functional clusters include Rho GTPase effectors, positive regulation of GTPase activity, regulation of cytoskeleton organization, cortical actin cytoskeleton organization, and septin complex (Fig. 2*D*). Additionally, protein interaction network analysis by Metascape revealed six protein complexes, which were annotated to be involved in cytoskeleton-dependent cytokinesis, protein neddylation, mRNA splicing, protein localization to organelle, Rho GTPase cycle, and signaling by Rho GTPases (Fig. 2*E* and supplemental Fig. S2*D*). Collectively, these analyses indicate that RID targets a broad range of proteins to change the *N*^ε^-fatty-acylation landscape of host cells and may induce widespread alterations in host cellular processes, especially in GTPase regulation, cytoskeletal organization, and cell division.

The substrates of RID were then compared with those of the *Shigella* effector IcsB (31). We found that the substrate scope of RID is much broader than that of IcsB. The Venn diagram showed that there is only a slight overlap between RID and IcsB substrates and that they both have their unique substrates (supplemental Fig. S3*A* and supplemental Table S3*A*). Notably, 93% of RID substrates are not modified by IcsB. As noted in the previous study (31) and confirmed in our bioinformatics analysis (supplemental Fig. S3*B* and supplemental Table S3*B*), the substrate proteins of IcsB are largely membrane-associated or within a membrane complex, and many are post-translationally modified by lipids in the forms of prenylation, *S*-palmitoylation, and *N*-myristylation (supplemental Fig. S3*C* and supplemental Table S3*B*). Moreover, protein class analysis showed that approximately 42% of IcsB substrates belong to small GTPases (supplemental Fig. S3*D*), indicating that IcsB has a strong substrate preference for these proteins. In comparison, RID appears to have no such substrate preference for lipidated proteins and small GTPases (supplemental Fig. S3, *C* and *D*). In addition, RID targets various classes of proteins with diverse cellular localizations including cytosol, actin cytoskeleton, midbody, and plasma membrane (Fig. 2*A* and supplemental Fig. S3*B*). Thus, RID has a substrate scope significantly different from IcsB.

### Septin Proteins are *N*^ε^-Fatty-Acylated on Multiple Lysine Residues in *C*-Terminal PBRs by RID

**Fig. 3:**
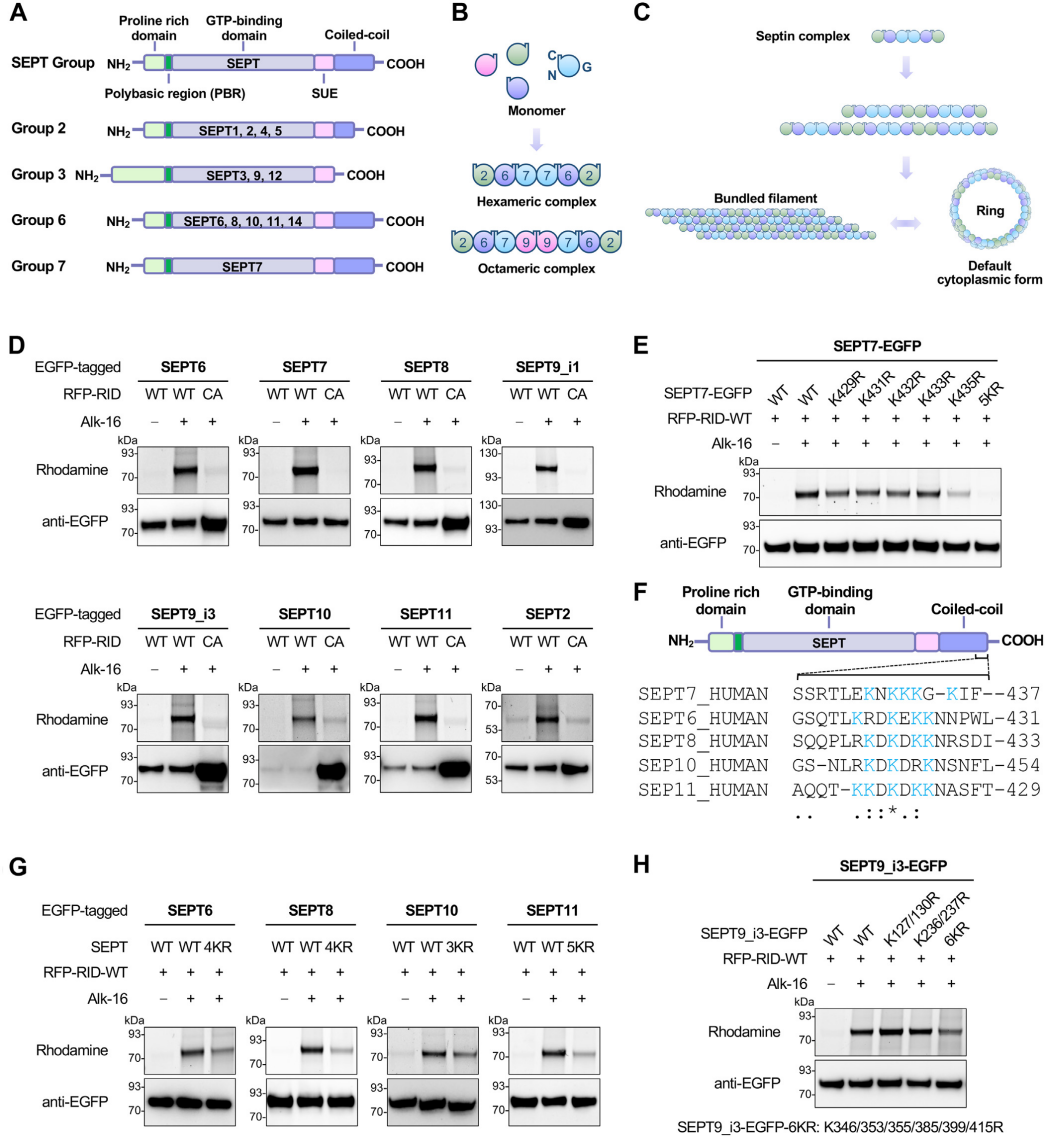
**Septin proteins are *N***^**ε**^**-fatty-acylated on multiple lysine residues by RID.***A*, schematic of septin structures and groups. The 13 human septins (SEPT1–SEPT12 and SEPT14) are classified into four groups (SEPT2, SEPT3, SEPT6, and SEPT7). *B*, schematic of the formation of SEPT2–SEPT6–SEPT7 and SEPT2–SEPT6–SEPT7–SEPT9 complexes. Septin subunits interact through their GTP-binding domain (G) and *N*-terminal and *C*-terminal regions (N and C, respectively) to form complexes. *C*, septin complexes can associate to form higher-order structures, including filaments and rings. Septins are in the default ring form and dynamically switch between linear bundles and cytoplasmic rings. *D*, validation of RID-mediated *N*^ε^-fatty-acylation of septin proteins. Septin proteins were individually co-transfected with RID-WT or RID-CA into HEK293T cells. The cells were metabolically labeled with Alk-16 and subjected to the in-gel fluorescence assay. Anti-EGFP immunoblotting is shown to confirm sample loading. *E*, effects of lysine mutation on RID-mediated *N*^ε^-fatty-acylation of SEPT7. *F*, alignment of *C*-terminal sequences of human septin proteins from the SEPT6 and SEPT7 groups. *C*-terminal lysine residues are shown in *blue*. *G*, effects of lysine mutation on RID-mediated *N*^ε^-fatty-acylation of SEPT6, SEPT8, SEPT10, SEPT11. *H*, effects of lysine mutation on RID-mediated *N*^ε^-fatty-acylation of SEPT9. For (*E*, *G*, and *H*), HEK293T cells were co-transfected with RID and individual septin proteins or the indicated lysine mutant, metabolically labeled with Alk-16, and subjected to in-gel fluorescence assay. Anti-EGFP immunoblotting is shown to confirm sample loading.

In bioinformatics analysis of the candidate substrates of RID, we noticed that a protein complex is annotated with a biological role in cytoskeleton-dependent cytokinesis and comprised of many septin proteins including SEPT6, SEPT7, SEPT8, SEPT9, SEPT10, and SEPT11 (Fig. 2*E* and Supplemental Fig. S4*A*). Septins constitute a family of highly conserved GTP-binding proteins that are associated with cellular membranes, actin filaments, and microtubules (51). Septins are recognized as the fourth component of the cytoskeleton (51) and play essential roles in many cellular processes ranging from cytokinesis (52) and exo/endocytosis (53) to host-pathogen interactions (54). The septin protein family consists of 13 members (SEPT1–SEPT12 and SEPT14) in humans from four groups, that is, SEPT2, SEPT3, SEPT6, and SEPT7 groups (Fig. 3*A*) (55). Human septins contain variable *N*-terminal and *C*-terminal regions and three conserved domains, that is, a PBR, a GTP-binding domain, and a septin unique element (SUE) (Fig. 3*A*) (55). In humans, septin monomers self-assemble predominantly into hetero-hexamers (SEPT2–SEPT6–SEPT7–SEPT7–SEPT6–SEPT2) (56, 57) or hetero-octamers (SEPT2–SEPT6–SEPT7–SEPT9–SEPT9–SEPT7–SEPT6–SEPT2) (58, 59) (Fig. 3*B*), with each member possibly substitutable for another member of the same group. A mixture of the hexameric and octameric complexes can further assemble into higher-order septin structures such as bundled filaments and circular rings (Fig. 3*C*) (51).

Considering the increasing recognition of the importance of septins in bacterial infection (60), we next sought to explore the RID-mediated *N*^ε^-fatty-acylation of septins. To minimize the interference from endogenous septins, we constructed a series of plasmids for expressing EGFP-tagged septins that were separable from endogenous counterparts in SDS-PAGE. As expected, using the Alk-16 labeling and in-gel fluorescence assay, we confirmed that representative septin members, including SETP6, SEPT7, SEPT8, SEPT9 (both isoforms 1 and 3, *i.e.*, SEPT9_i1 and SEPT9_i3), SEPT10, and SEPT11, were indeed strongly *N*^ε^-fatty-acylated in a RID-dependent manner (Fig. 3*D*), further validating the proteomics data. It is worth mentioning that SEPT2 was identified in the proteomics data with low SILAC ratios (supplemental Table S1) and thus was only weakly modified by RID as shown in Figure 3*D*. Consistent with these results, we showed that RID-CA was associated with septin proteins of different groups *via* a co-immunoprecipitation (co-IP) assay (supplemental Fig. S4*B*).

The previous study has shown that IcsB-catalyzed *N*^ε^-fatty-acylation occurs on lysine residues in PBRs of substrate proteins (31). We therefore initially examined the conserved PBR in septin proteins (Fig. 3*A*) to map the modification sites. However, mutation of lysines (*i.e.*, K43 and K46) in the *N*-terminal PBR of SEPT7 (61), which belongs to the SEPT7 group, into arginines or mutation of all basic residues in this region into alanines failed to decrease Alk-16 labeling intensities (supplemental Fig. S4*C*). By contrast, we were delighted to find that deletion of the *C*-terminal disordered region in the coiled-coil domain (from residue 378 to 437) of SEPT7 abolished RID-mediated *N*^ε^-fatty-acylation (supplemental Fig. S4C). It was noted that a PBR (*i.e.*, ^428^EKNKKKGK^435^) was present at the very end of the *C*-terminus of SEPT7. To further determine the exact modification sites of SEPT7, we thus focused on its *C*-terminal PBR that contains multiple lysines and generated a series of lysine-to-arginine mutants (Fig. 3, *E* and *F*). While single-residue mutation (*i.e.*, K429R, K431R, K432R, K433R, and K435R) reduced the modification levels of SEPT7 to varying extents, the quintuple mutant (*i.e.*, 5KR; K429/431/432/433/435R) showed a completely diminished *N*^ε^-fatty-acylation in the presence of RID (Fig. 3*E*). Sequence alignment showed that SEPT6 group members such as SEPT6, SEPT8, SEPT10, and SEPT11 contain similar PBRs and multiple lysines in their *C*-terminal tails that are conserved with those in SEPT7 (Fig. 3*F*). Similarly, mutation of these lysines in the *C*-terminal PBRs of SEPT6, SEPT8, SEPT10, and SEPT11 significantly decreased their *N*^ε^-fatty-acylation levels by RID (Fig. 3*G*). In the case of SEPT9, this septin member belongs to the SEPT3 group and has no conserved *C*-terminal coiled-coil domain (Fig. 3*A*) (55). We found that RID-mediated *N*^ε^-fatty-acylation of SEPT9 isoform 3, *i.e.*, SEPT9_i3, occurred more promiscuously on multiple lysines near the *C*-terminus of the protein, as mutation of these lysines (*i.e.*, K346, K353, K355, K385, K399, and K415), but not those in the middle of SEPT9 (*i.e.*, K127, K130, K236, and K237), reduced the modification level (Fig. 3*H*). Notably, although SEPT2 contains a *C*-terminal coiled-coil region (*i.e.*, residues 317–346) (55) (Fig. 3*A*), there are no obvious polybasic sequences in this region and only sparse lysines (*i.e.*, K318 and K325) are present. Thus, SEPT2 was much less modified by RID in comparison with septin proteins of SEPT6 and SEPT7 groups (Fig. 3*D*). Taken together, RID mediates *N*^ε^-fatty-acylation of a series of septin proteins, especially the members from SEPT6 and SEPT7 groups including SEPT6, SEPT8, SEPT10, SEPT11, and SEPT7, on multiple lysines in their *C*-terminal PBRs of the coiled-coil regions.

### RID-Mediated *N*^ε^-Fatty Acylation Disrupts the Assembly and Organization of Septin Filaments

**Fig. 4:**
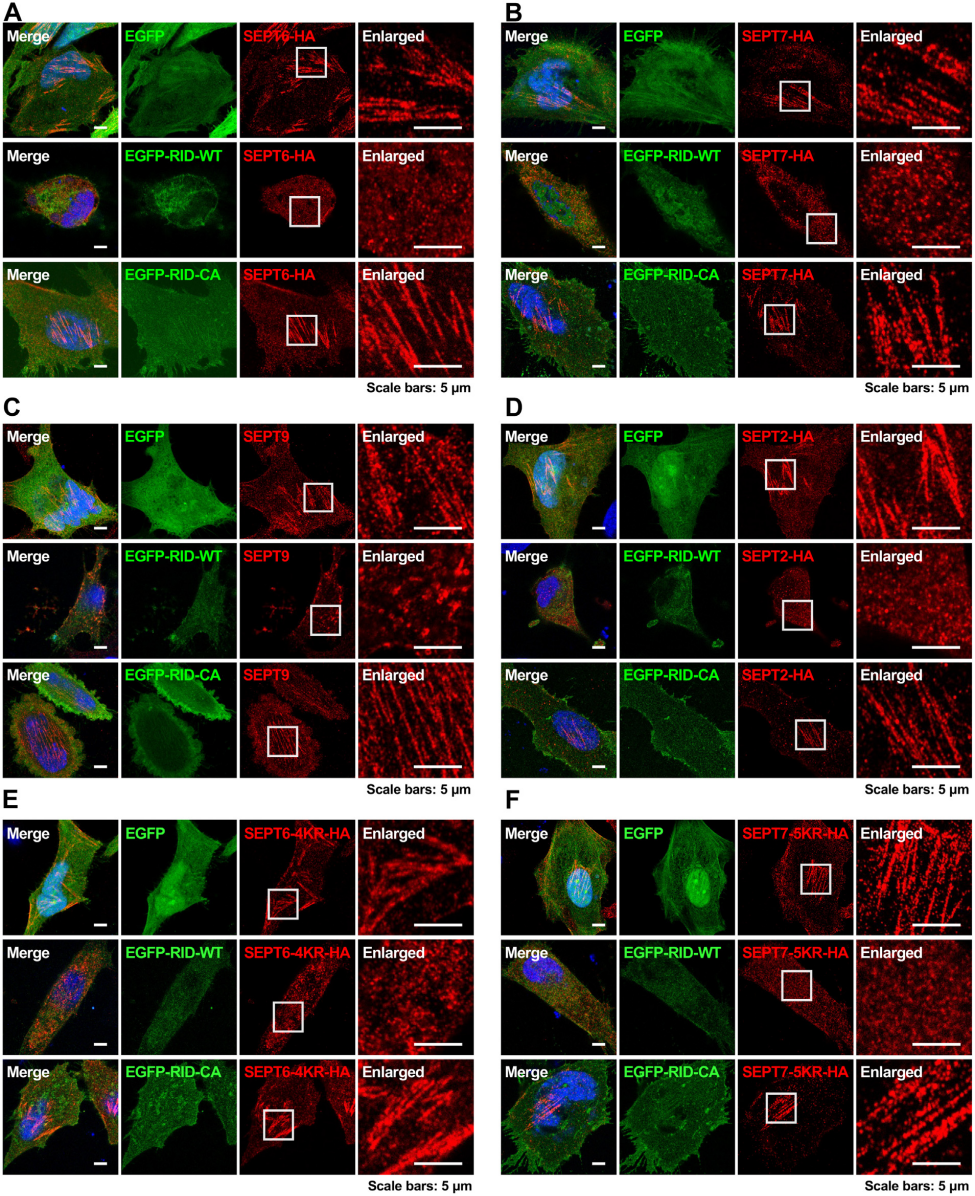
**RID expression disrupts the assembly of septin filaments.***A*, immunofluorescence imaging of SEPT6 in the absence and presence of RID activity. *B*, immunofluorescence imaging of SEPT7 in the absence and presence of RID activity. *C*, immunofluorescence imaging of SEPT9 in the absence and presence of RID activity. *D*, immunofluorescence imaging of SEPT2 in the absence and presence of RID activity. *E*, immunofluorescence imaging of SEPT6-4KR in the absence and presence of RID activity. *F*, immunofluorescence imaging of SEPT7-5KR in the absence and presence of RID activity. For (*A, B, D, E,* and *F*), HeLa cells were co-transfected with individual HA-tagged septin proteins or mutants and RID or the inactive mutant and processed for anti-HA immunostaining. For (*C*), HeLa cells were transfected with RID or the inactive mutant and processed for anti-SEPT9 immunostaining. The enlarged images show the magnified areas in the *white rectangles*.

To explore the effects of RID-mediated *N*^ε^-fatty-acylation on septin proteins in mammalian cells, we performed immunofluorescence imaging experiments. For this purpose, HeLa cells were transfected with HA-tagged SEPT6 and EGFP-tagged RID-WT or RID-CA for immunostaining. In cells transfected with EGFP vector or the inactive RID-CA mutant, SEPT6 clearly assembled into filamentous structures (Fig. 4*A*) as previously reported (62, 63), consistent with the notion that septins associate together to form higher-order filaments (51). By contrast, such filaments were disrupted upon RID-WT expression. Specifically, we observed that SEPT6 formed highly diffused intracellular puncta in RID-WT-expressing cells (Fig. 4*A*). We then examined whether the organization of other septins was affected by RID and found that the filamentous structures formed by overexpressed SEPT7 and endogenous SEPT9 were also disrupted in RID-WT-expressing cells like that observed for SEPT6 (Fig. 4, *B* and *C*). The filamentous structures of the less modified SEPT2 were altered by RID as well (Fig. 4*D*). In agreement with this result, despite the much less RID-mediated modifications of SEPT6 quadruple lysine-to-arginine mutant and SEPT7 quintuple mutant (*i.e.*, SEPT6-4KR and SEPT7-5KR, respectively) (Fig. 3, *E* and *G*), the organization of SEPT6-4KR and SEPT7-5KR was also unexpectedly perturbed upon RID expression, changing from fibrillar structures to diffused puncta (Fig. 4, *E* and *F*). To understand this, we showed that both HA-tagged SEPT6 and its quadruple mutant SEPT6-4KR strongly colocalized with endogenous SEPT9 (supplemental Fig. S5*A*) and interacted with other endogenous septin proteins (*e.g.*, SEPT2, SEPT7, and SEPT9) (supplemental Fig. S5*B*). Similarly, we found that HA-tagged SEPT7 and its quintuple mutant SEPT7-5KR colocalized with endogenous SEPT9 and interacted with endogenous septin proteins (*e.g.*, SEPT2, SEPT6, and SEPT9) (supplemental Fig. S5, *C* and *D*). These observations are notably consistent with a previous study showing HA-tagged SEPT6 and SEPT7 colocalize with endogenous septins (64). Therefore, we speculate that one reason behind the observed perturbation of SEPT6-4KR and SEPT7-5KR organization upon RID expression is probably because endogenous septin proteins were *N*^ε^-fatty-acylated by RID and the overall septin structures were disrupted, compelling organization changes of SEPT6-4KR and SEPT7-5KR that have been incorporated into endogenous septin complexes. Nevertheless, RID appears to prevent the assembly and organization of septin proteins into higher-order filamentous structures through its *N*^ε^-fatty-acylation activity.

### RID-Mediated *N*^ε^-Fatty Acylation Does Not Affect Septin–Septin Interactions

**Fig. 5:**
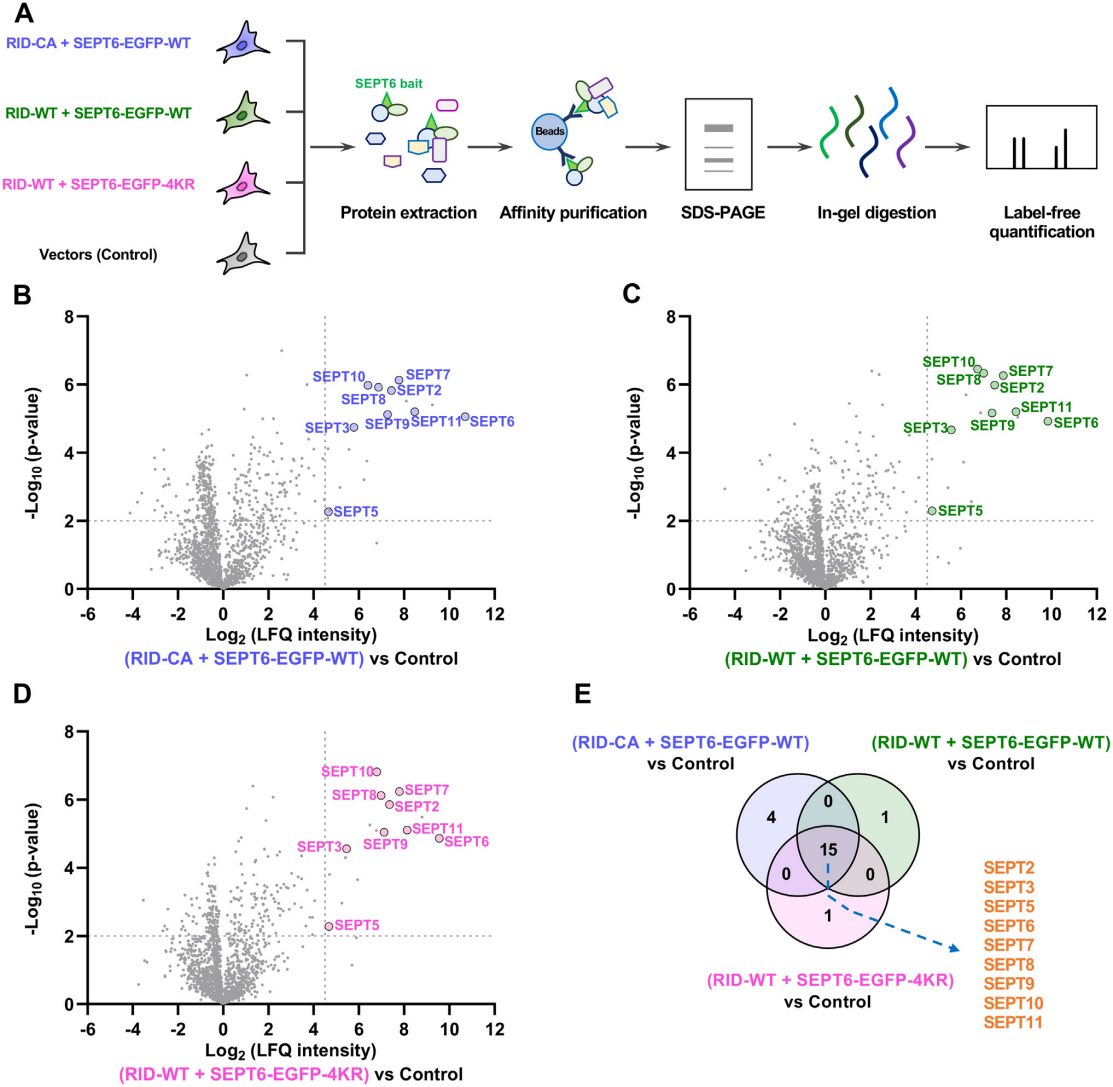
**RID-mediated *N***^**ε**^**-fatty-acylation does not affect septin interactions.***A*, schematic for affinity purification and mass spectrometry analysis of SEPT6 interacting proteins in the absence and presence of RID-mediated *N*^ε^-fatty-acylation. HEK293T cells were co-transfected with the indicated plasmids, using corresponding vectors without RID and SEPT6 inserts as the control, and processed in three biological replicates for affinity purification, in-gel digestion, proteomic identification, and LFQ. *B*, volcano plot of the LFQ proteomics data for affinity purification of SEPT6 in the presence of RID-CA. Protein hits with significant enrichment, *i.e.*, log_2_ (LFQ intensity) >4.5 and −log_10_ (*p*-value) >2, in the SEPT6-WT- and RID-CA-expressing samples relative to the control samples are considered as reliable SEPT6-WT interacting proteins. *C*, volcano plot of the LFQ proteomics data for affinity purification of SEPT6 in the presence of RID-WT. Protein hits with significant enrichment, *i.e.*, log_2_ (LFQ intensity) >4.5 and −log_10_ (*p*-value) >2, in the SEPT6-WT- and RID-WT-expressing samples relative to the control samples are considered as reliable SEPT6-WT interacting proteins in the presence of RID. *D*, volcano plot of the LFQ proteomics data for affinity purification of SEPT6-4KR in the presence of RID-WT. Proteins hits with significant enrichment, *i.e.*, log_2_ (LFQ intensity) >4.5 and −log_10_ (*p*-value) >2, in the SEPT6-4KR- and RID-WT-expressing samples relative to the control samples are considered as reliable SEPT6-4KR interacting proteins. *E*, Venn diagram showing overlap of SEPT6-WT and SEPT6-4KR interacting proteins in the presence or absence of RID activity. Protein hits with significant enrichment in (*B*–*D*) are used for the Venn diagram.

Septin proteins interact intermolecularly to form hetero-oligomeric complexes that further assemble into higher-order filaments (51, 55). To investigate whether the interactions among septin proteins are affected by RID, we conducted affinity purification-mass spectrometry (AP-MS) experiments. In this regard, EGFP-tagged SEPT6 or its quadruple mutant SEPT6-4KR was co-expressed with RID-WT or RID-CA in cells and immunoprecipitated by anti-EGFP beads. The resulting co-immunoprecipitates were analyzed and compared with the vector control by label-free quantification (LFQ) proteomics (43) to identify and quantify septin proteins associated with SEPT6 (Fig. 5*A*). The MS analysis showed that SEPT6 was indeed co-affinity-purified with endogenous septin proteins, such as SEPT2, SEPT3, SEPT5, SEPT7, SEPT8, SEPT9, SEPT10, and SEPT11, in the absence of RID activity (Fig. 5*B* and supplemental Table S4). More importantly, the interactions between SEPT6 and these septins were not affected by RID-WT expression (Fig. 5*C* and supplemental Table S4). Interestingly, the SEPT6-4KR mutant could still interact and likely form heteromeric complexes with other endogenous septin proteins that were *N*^ε^-fatty-acylated by RID (*e.g.*, SEPT2, SEPT3, SEPT5, SEPT6, SEPT7, SEPT8, SEPT9, SEPT10, and SEPT11) in the presence of RID activity (Fig. 5*D* and supplemental Table S4). This result provides further support to the above speculation for the observation that the organization of SEPT6-4KR was perturbed in RID-WT-expressing cells (Fig. 4*E*), as the SEPT6-4KR mutant still associates with disrupted endogenous septin structures. Overall, the septin-septin hetero-interactions were quantitatively retained in these conditions (Fig. 5*E* and supplemental Fig. S6*A*). These results were further confirmed by Western blot analysis of the anti-EGFP co-immunoprecipitates using antibodies against representative endogenous septin proteins (supplemental Fig. S6*B*). Together, these studies indicate that RID-mediated *N*^ε^-fatty-acylation does not alter the lower-order oligomeric interactions of septin proteins.

### RID-Mediated *N*^ε^-Fatty Acylation Confers Localization of Septin Proteins to DRMs

**Fig. 6:**
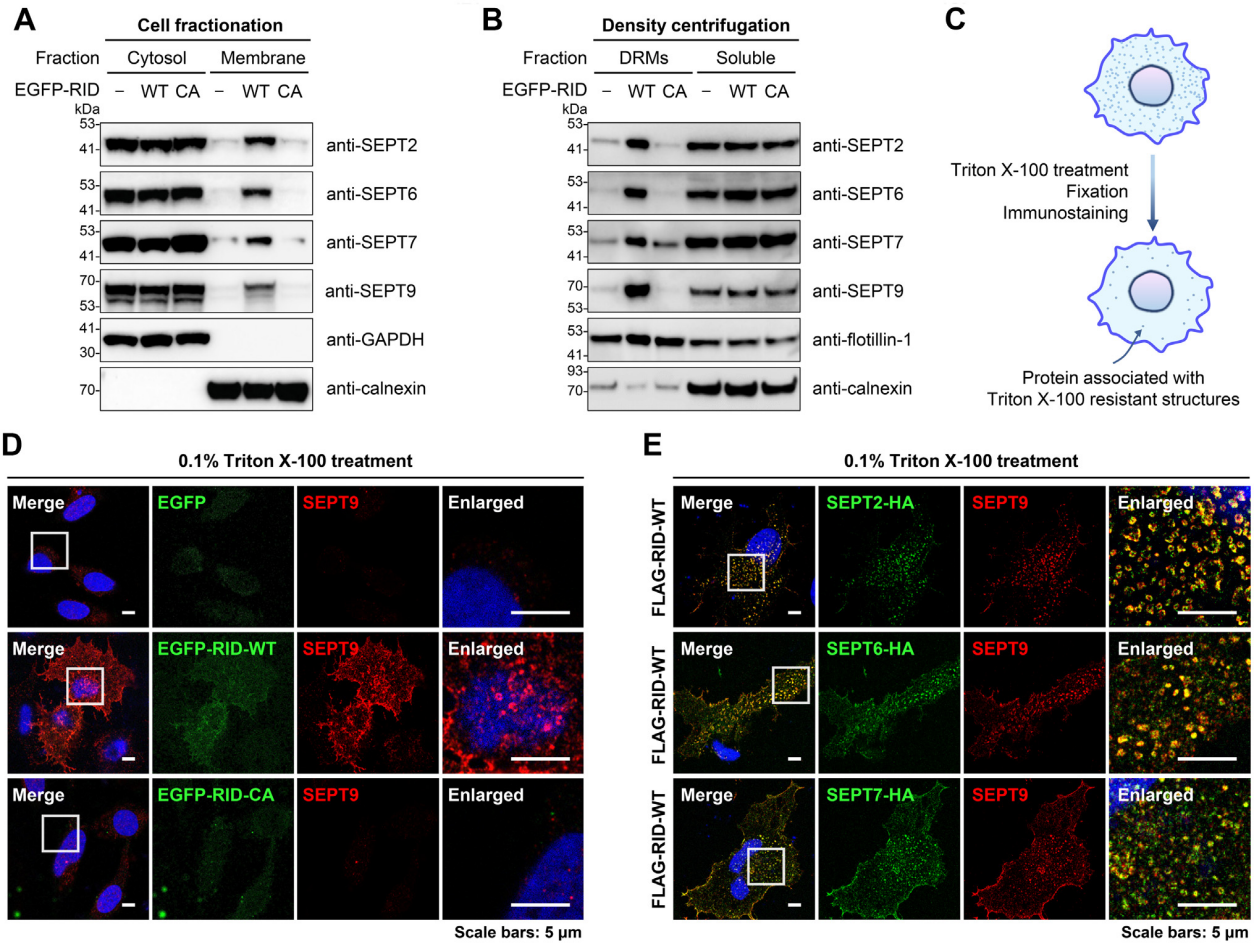
**RID-mediated *N***^**ε**^**-fatty-acylation increases membrane affinity of septin proteins and confers localization to detergent-resistant membranes.***A*, cell fractionation assay showing the effects of RID-mediated *N*^ε^-fatty-acylation on membrane affinity of septin proteins. Cells expressing RID-WT or RID-CA were lysed and processed to separate the cytosol and membrane fractions. *B*, protein flotation assay to analyze the localization of septin proteins to DRMs upon RID-mediated *N*^ε^-fatty-acylation. Cells expressing RID-WT or RID-CA were lysed and processed by the protein flotation assay to separate the DRMs and soluble proteins. *C*, schematic for immunofluorescence analysis of proteins associated with TX100-resistant structures. *D*, immunofluorescence imaging of the organization of endogenous SEPT9 after TX100 treatment in the absence and presence of RID activity. HeLa cells were transfected with RID or the inactive mutant, pre-treated with TX100, and processed for anti-SEPT9 immunostaining. *E*, immunofluorescence imaging of the organization of septin proteins after TX100 treatment in the presence of RID activity. HeLa cells were co-transfected with individual HA-tagged septin proteins and RID, pre-treated with TX100, and processed for anti-HA and SEPT9 immunostaining. The enlarged images show the magnified areas in the *white rectangles*.

Considering the high hydrophobicity of long-chain fatty-acyl groups (21, 40), we proceeded to examine whether RID-mediated *N*^ε^-fatty-acylation could affect the membrane affinity and localization of septins. For this, cells expressing RID-WT or RID-CA were lysed and the lysates were fractionated to separate cytosol and membrane proteins. Western blot analysis of the cytosol and membrane fractions showed that representative *N*^ε^-fatty-acylated septins such as SEPT2, SEPT6, SEPT7, and SEPT9 from RID-WT-expressing cells were significantly more localized in the membrane fraction, compared to those from mock- or RID-CA-transfected cells (Fig. 6*A*), indicating that RID-mediated *N*^ε^-fatty-acylation enhances the hydrophobicity and membrane affinity of septins.

Lipid modifications with saturated acyl chains such as *N*-myristoylation and *S*-palmitoylation are known to target proteins to DRM rafts (65, 66), which are membrane domains rich in sphingolipid and cholesterol and insoluble in non-ionic detergents (67). We therefore tested whether RID-mediated *N*^ε^-fatty-acylation changes the intracellular localization of septin proteins to DRMs. To this end, DRMs were isolated from other membrane domains using the protein flotation assay by lysing cells with non-ionic detergent Triton X-100 (TX100) and separating fractions by density centrifugation in OptiPrep (41). Notably, we observed that expression of RID-WT, but not EGFP vector or RID-CA mutant, led to a significant enrichment of septin proteins (*e.g.*, SEPT2, SEPT6, SEPT7, and SEPT9) in DRMs that are characterized by flotillin-1 as a DRM marker (Fig. 6*B*). These results thus suggest that long-chain *N*^ε^-fatty-acylation may serve as a DRM targeting signal.

A distinct feature of DRMs is the resistance to solubilization with TX100 (68, 69). Hence to further corroborate the detergent-resistance of septins and probe the detailed organization of detergent-resistant septins in the presence of RID, we performed additional immunofluorescence imaging experiments in which the cells were pre-treated with 0.1% TX100 at 4 °C to release detergent-soluble proteins before fixation and immunostaining (45) (Fig. 6*C*). The imaging analyses showed that endogenous SEPT9 in RID-WT-expressing cells was largely retained after TX100 treatment, whereas the SEPT9 signals in RID-CA-expressing cells were significantly diminished (Fig. 6*D*), confirming the protein flotation assay results showing that SEPT9 became resistant to TX100 solubilization upon *N*^ε^-fatty-acylation by RID (Fig. 6*B*). In addition, we observed that other septins (*e.g.*, SEPT2, SEPT6, and SEPT7) were similarly Triton-resistant in RID-WT-expressing cells and well-colocalized with SEPT9 (Fig. 6*E*), consistent with the above observation in the AP-MS experiments showing that septin proteins were associated together. Thus, these data suggest that RID-mediated *N*^ε^-fatty-acylation increases the membrane affinity of septins and confers localization of septin proteins to DRMs.

### RID-Mediated *N*^ε^-Fatty Acylation Induces the Formation of Triton-Resistant Septin Rings and Alters Septin Dynamics Between Filaments and Rings

**Fig. 7:**
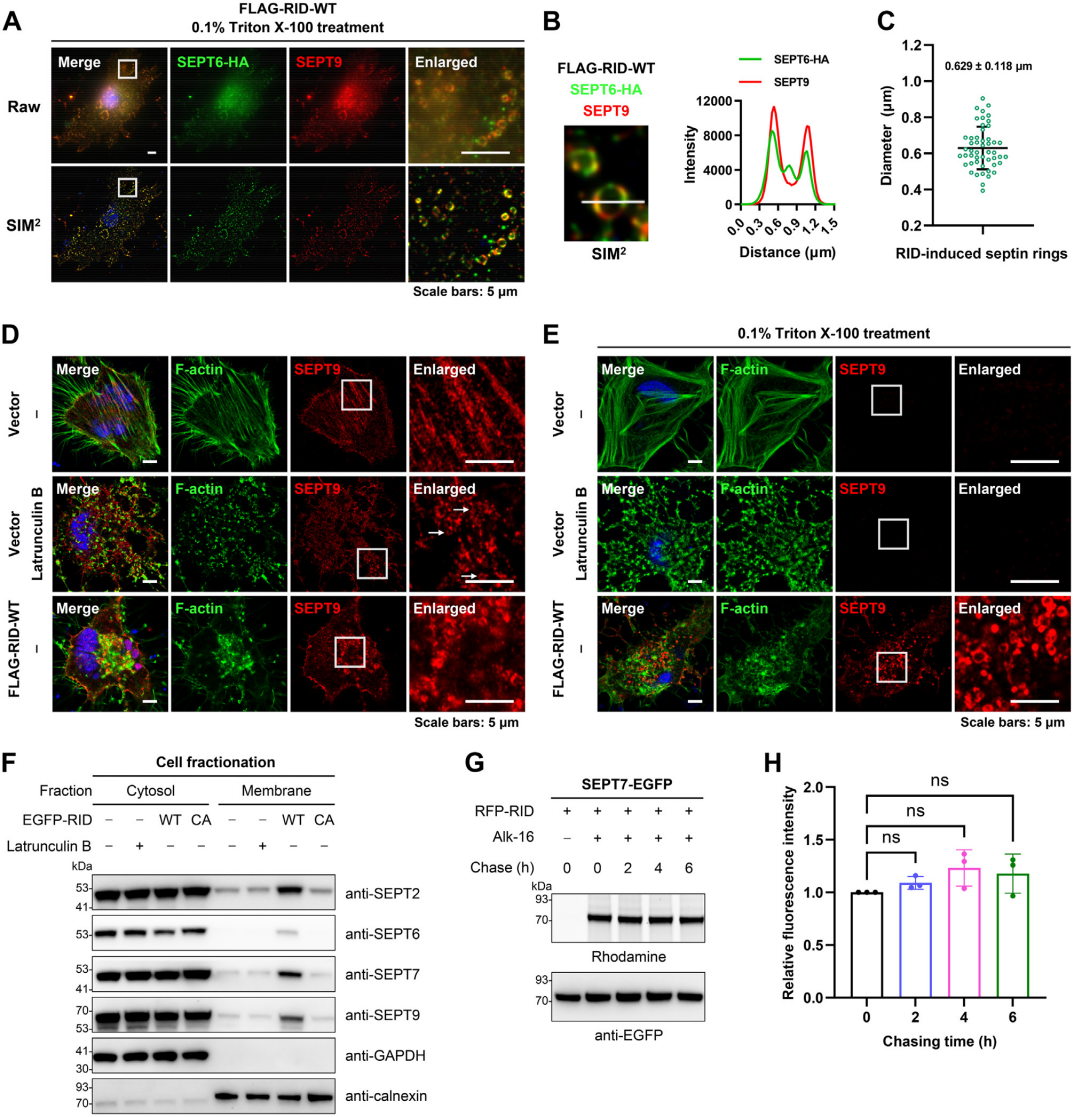
**RID-mediated *N***^**ε**^**-fatty-acylation induces the formation of Triton-resistant septin rings.***A*, super-resolution SIM^2^ imaging of the organization of septin proteins after TX100 treatment in the presence of RID activity. HeLa cells were co-transfected with HA-tagged SEPT6 and RID, pre-treated with TX100, and processed for anti-HA and anti-SEPT9 immunostaining. The enlarged images show the magnified areas in the *white rectangles*. *B*, the magnified septin rings observed in the SIM^2^ image shown in (*A*) and the fluorescence intensity profiles of SEPT6 and SEPT9 in the septin ring. *C*, the statistical analysis of diameters of RID-induced septin rings (*n* = 50). *D*, comparison of septin rings induced by RID expression with those induced by latrunculin B without TX100 pretreatment. HeLa cells were transfected with RID, incubated with or without latrunculin B, and processed for anti-SEPT9 immunostaining and F-actin staining with phalloidin. The enlarged images show the magnified areas in the *white rectangles*. The *white arrows* show the septin rings formed upon actin collapse. *E*, comparison of septin rings induced by RID expression with those induced by latrunculin B after TX100 pretreatment. HeLa cells were processed as in (*D*) unless pretreatment with TX100 before fixation and immunostaining. The enlarged images show the magnified areas in the *white rectangles*. *F*, cell fractionation assay to compare the effects of RID-mediated *N*^ε^-fatty-acylation and latrunculin B on membrane affinity of septin proteins. Cells incubated with latrunculin B or transfected to express RID-WT or RID-CA were lysed and processed to separate the cytosol and membrane fractions. *G*, pulse-chase assay to analyze the turnover of RID-mediated *N*^ε^-fatty-acylation of SEPT7. Cells expressing RID-WT and SEPT7 were metabolically labeled with Alk-16 for 10 h, chased with palmitic acid for indicated periods, and subjected to in-gel fluorescence assay. Anti-EGFP immunoblotting is shown to confirm sample loading. *H*, quantification of pulse-chase Alk-16 labeling of SEPT7 shown in (*G*). Fluorescence intensities of SEPT7 bands were quantified and normalized. Data are represented as mean ± s.d., *n* = 3, and ns indicates a *p*-value >0.05, calculated by one-way ANOVA test.

To probe the sub-resolution organization of detergent-resistant septins, we performed super-resolution structured illumination microscopy (SIM) on RID-expressing cells that were co-stained for SEPT6 and SEPT9 (Fig. 7*A*). Strikingly, we observed that both exogenous SEPT6 and endogenous SEPT9 formed Triton-resistant ring-like structures with diameters of 0.629 ± 0.118 μm (mean ± sd, *n* = 50) and that these septin proteins strongly colocalized on these rings (Fig. 7, *B* and *C*).

The septin complexes are known to adopt higher-order filamentous structures colocalizing with actin stress fibers in cells (58, 62, 70) but have an inherent tendency to self-assemble into rings that represent a default storage form and a cytoplasmic pool of septins (44, 58, 62). When the actin architecture is disrupted by chemical agents such as latrunculin B or cytochalasin D, septin organization changes from filaments to ring structures of around 0.6 μm diameters (62), which are similar to those of RID-induced septin rings. Within 30 min after the washout of actin-disrupting agents, the ring formation can be reversed with the restoration of septin filaments. We thus asked whether the septin rings formed upon actin disruption are different from the ring-like structures induced by RID. Our immunofluorescence imaging showed that treatment of cells with latrunculin B indeed led to the collapse of actin fibers, disruption of septin filaments, and formation of actin-independent septin rings as previously reported (44, 58, 62) (Fig. 7*D*). However, these septin rings were not resistant to detergent and could be efficiently removed by TX100 extraction, whereas the ring-like structures formed in RID-WT-expressing cells were consistently resistant to Triton solubilization (Fig. 7*E*). In addition, the cell fractionation assay revealed that the membrane association of septin proteins was not affected by latrunculin B (Fig. 7*F*). Thus, the actin-independent septin rings induced by latrunculin B are likely in the cytoplasm and not associated with DRMs.

To explore the stability of RID-induced septin rings, we analyzed the stability of RID-mediated *N*^ε^-fatty-acylation using pulse-chase labeling experiments. Specifically, the cells were pulse-labeled with Alk-16, chased with a large excess of palmitic acid, and processed for the in-gel fluorescence assay. As shown in Figure 7, *G* and *H*, the Alk-16 labeling of SEPT7 remained unchanged within several hours, indicating that RID-mediated *N*^ε^-fatty-acylation is relatively stable in cells. Therefore, we suggest that the resulting ring-like structures of septins may not be readily reversed. Overall, the septin ring-like structures induced by RID-mediated *N*^ε^-fatty-acylation are substantially different from septin rings observed upon actin collapse in many aspects such as detergent resistance, localization, membrane affinity, and reversibility. Considering that the septin organization in mammalian cells is dynamic and switches between actin-bundled filaments and cytoplasmic rings as a storage form (58, 62, 70), we propose that RID-mediated *N*^ε^-fatty-acylation stably targets and sequesters septin proteins to DRM rafts as Triton-resistant septin rings to limit the cytoplasmic supply of septins from assembling into filaments, thereby resulting in altered septin dynamics and filamentous organization.

### RID-Mediated *N*^ε^-Fatty Acylation Impairs the Cytoskeleton, Cytokinesis, and Cell Proliferation

**Fig. 8:**
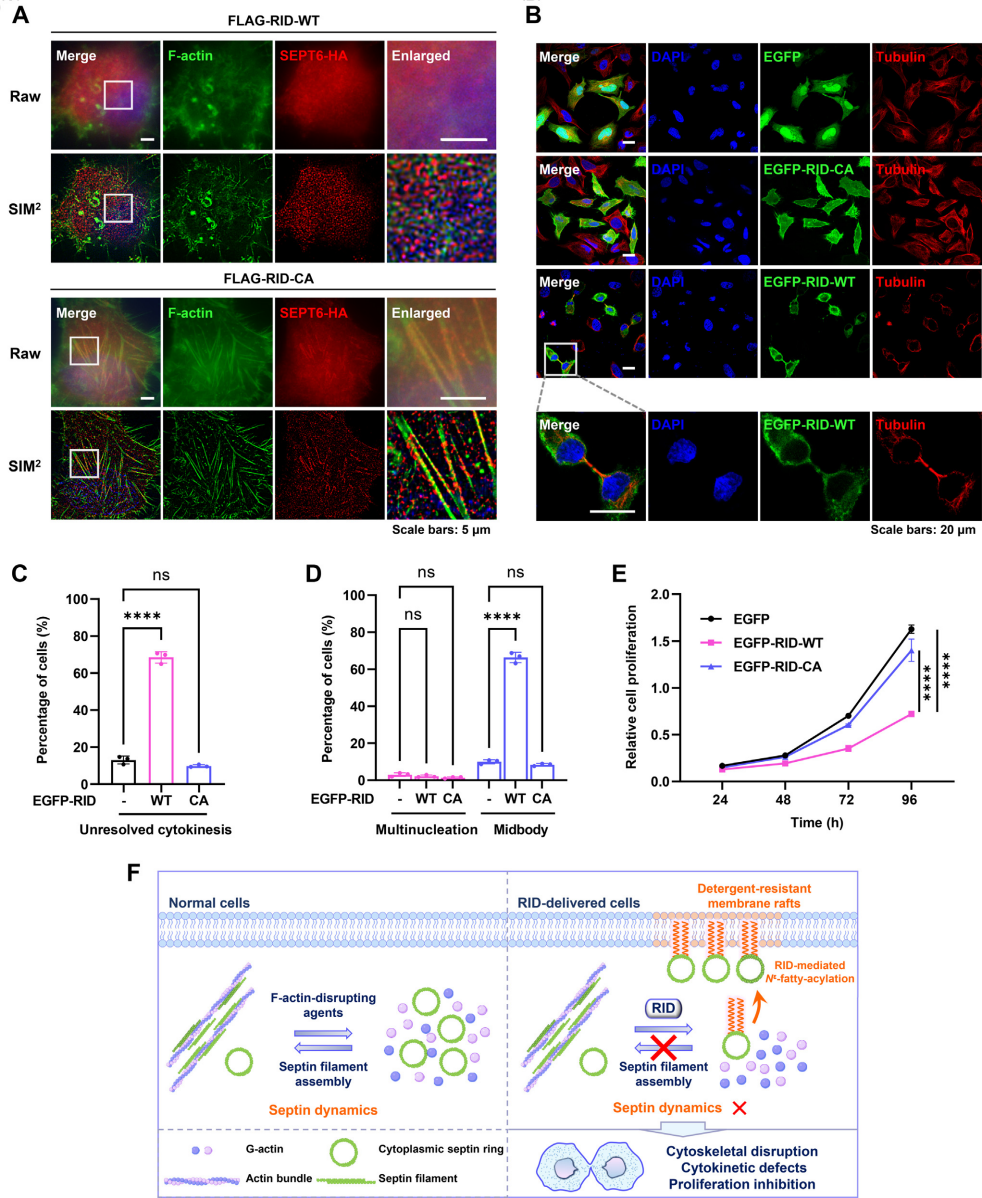
**RID-mediated *N***^**ε**^**-fatty-acylation causes defects in the actin cytoskeleton, cytokinesis, and cell proliferation.***A*, fluorescence imaging of the actin cytoskeleton in the presence and absence of RID activity. HeLa cells were co-transfected with HA-tagged SEPT6 and RID or its inactive mutant and processed for anti-HA immunostaining and F-actin staining with phalloidin. The enlarged images show the magnified areas in the *white rectangles*. *B*, fluorescence imaging analysis of cytokinesis and the tubulin organization in the presence and absence of RID activity. HeLa cells were transfected with RID or its inactive mutant and processed for anti-tubulin immunostaining. The enlarged images show the magnified areas in the *white rectangles*. *C*, quantification of cells with unresolved cytokinesis in fluorescence images shown in (*B*). *D*, quantification of cells with multinucleation and midbody in fluorescence images shown in (*B*). *E*, cell proliferation assay to analyze the effects of RID-WT and its inactive mutant on cell growth. HEK293T cells were transfected with RID or its inactive mutant and incubated for indicated periods before cell viability assay. Quantification data are represented as mean ± s.d., *n* = 3. ∗∗∗∗ indicates a *p*-value <0.0001 and ns indicates a *p*-value >0.05, calculated by one-way ANOVA test. *F*, the proposed model in which RID-mediated *N*^ε^-fatty-acylation of septins targets septin proteins to DRM rafts and therefore affects septin organization and dynamics, further contributing to cytoskeletal disruption and cytokinetic defects.

Previous studies have shown that septins play an essential role in actin assembly and stabilization (62, 70, 71, 72). When septins are sequestered or depleted, actin bundles are destabilized and the spatial organization is disrupted (58, 73, 74). Our confocal and super-resolution SIM imaging analyses demonstrated that the expression of RID-WT, but not RID-CA, led to attenuation of actin bundles in HeLa cells (Fig. 8*A* and supplemental Fig. S7, *A*–*C*). Notably, these results phenocopy the effects of septin depletion by siRNA or septin sequestration by expression of septin-interacting domains in causing loss of actin bundles (62) and are in accordance with the sequestration of septins in DRMs and disruption of septin organization by RID-mediated *N*^ε^-fatty-acylation of septins. To examine the possibility of actin modification by RID, we performed the Alk-16 labeling and in-gel fluorescence assay and found that actin was not *N*^ε^-fatty-acylated by RID (supplemental Fig. S7*D*), suggesting that RID is unlikely to have a direct effect on actin organization. Therefore, RID indirectly affects the actin cytoskeleton probably through multiple pathways, such as the canonical inactivation of Rho family small GTPases (19) and *N*^ε^-fatty-acylation of septins resulting in disruption of septin organization.

Given that septins have important functions in cytokinesis (58, 75) and RID can change septin organization, we proceeded to examine whether RID could cause cytokinetic defects. As expected, expression of RID-WT, but not EGFP vector or RID-CA mutant, in HeLa cells led to an arrest at the late cytokinesis and abscission step, leaving persistent midbodies shown in the fluorescence images (Fig. 8*B*). Quantification of these images suggested that more than 70% of RID-WT-expressing cells were defective in cytokinesis (Fig. 8*C*). Interestingly, we observed that the percentage of multinucleated cells was not affected by RID and that 66.3% of RID-WT-expressing cells exhibited midbody attachment compared to only 8.4% of EGFP- or RID-CA-expressing cells attached with midbodies (Fig. 8*D*), demonstrating a significant increase of midbody-connected cells in the presence of RID. As a result of these defects in cell division, the proliferation of RID-WT-expressing cells was significantly suppressed (Fig. 8*E*). Notably, these data are consistent with previous studies that demonstrated the effects of septin depletion in causing cytokinetic defects and an increase of cells joined by midbodies (52, 58, 75, 76, 77). Taken together, these results suggest that RID-mediated *N*^ε^-fatty-acylation of septins and septin organization disruption are concomitant with defects in the actin cytoskeleton, late stages of cytokinesis, and cell growth.

## Discussion

In this study, we undertook a chemical proteomics approach to globally profile the *N*^ε^-fatty-acylation substrates of RID. This approach featuring bioorthogonal labeling of *N*^ε^-fatty-acylated proteins and SILAC-based quantification has allowed the reliable identification of a large number of previously unknown substrate proteins targeted by RID for the first time. Previous studies have shown that RID modifies Rho GTPases and inhibits their activation through *N*^ε^-fatty-acylation (19). While *N*^ε^-fatty-acylation of Rho GTPases contributes to the well-known function of RID in causing the cell rounding phenotype, the proteomic discovery of many other targets indicates that this effector domain broadly alters the *N*^ε^-fatty-acylation landscape of host proteins and thus may have many undetermined functions in modulating host cellular processes. Nevertheless, how RID-mediated *N*^ε^-fatty-acylation impacts individual target proteins largely remains to be investigated.

Among the numerous newly identified RID targets, we focused on septin proteins as most of the septin family members were retrieved to be potentially modified by RID in our quantitative chemical proteomic profiling. Studies have shown that the assembly and disassembly of septin filaments are controlled by PTMs (78), such as phosphorylation (79), sumoylation (64), and acetylation (80). We validated that RID can indeed catalyze the post-translational *N*^ε^-fatty-acylation modifications of most septin family members promiscuously on multiple lysines in *C*-terminal PBRs of the disordered regions to disrupt the organization of septin filaments. Further experiments showed that RID-mediated *N*^ε^-fatty-acylation not only increases the membrane affinity of septins but also confers triton-resistance and localization to DRMs. Using super-resolution fluorescence imaging, we found that septin proteins form ring-like structures that became resistant to extraction with TX100 and appeared to be not readily reversible, owing to the stable RID-mediated *N*^ε^-fatty-acylation of septins. We thus propose that these triton-resistant RID-induced ring-like structures of septins sequester the proteins in DRMs and reduce the relative abundance of default cytoplasmic septin rings. As a result, the dynamic switch between filamentous septins and cytoplasmic septin rings is compromised and the organization of septin filaments is disrupted in RID-expressing cells, which could further contribute to the collapsed actin cytoskeleton, cytokinetic defects, and suppressed cell proliferation (Fig. 8*F*). Notably, these phenotypes resulted from RID-mediated septin *N*^ε^-fatty-acylation resemble those reported in septin depletion studies (52, 74, 81) and are in accordance with the hypothesis that the cytoplasmic pool of septins is disrupted by RID-mediated *N*^ε^-fatty-acylation.

While we were able to locate RID-mediated *N*^ε^-fatty-acylation lysine residues in septins, the immunofluorescence imaging analysis showed that the filaments formed by the much less modified lysine-to-arginine mutants of SEPT6 and SEPT7 were also disrupted by RID to become puncta (Fig. 4, *E* and *F*). Our speculation would be that SEPT6-4KR and SEPT7-5KR are incorporated into the endogenous septin structures that can be disrupted by RID-mediated *N*^ε^-fatty-acylation and impact the observed patterns of SEPT6-4KR and SEPT7-5KR. In support of this, previous studies (64) and our analyses both revealed that SEPT6 and SEPT7, as well as the SEPT6-4KR and SEPT7-5KR mutants, colocalized and interacted with endogenous septins (supplemental Fig. S5). Moreover, the quantitative AP-MS and co-immunoprecipitation experiments demonstrated that the SEPT6-4KR mutant was associated with *N*^ε^-fatty-acylated endogenous septins in disrupted septin structures (Fig. 5 and supplemental Fig. S6), lending further support to the above speculation that the aberrant endogenous septin structures may affect the organization of SEPT6-4KR and SEPT7-5KR.

Previous studies have shown that RID modifies and inactivates known master regulators of actin bundles, that is, Rho family small GTPases such as RAC1, to disrupt the host actin cytoskeleton and induce cell rounding (19). Our present work confirms that Rho GTPases are modified by RID under complex cellular contexts. In another aspect, our work demonstrates that RID extensively *N*^ε^-fatty-acylates septin proteins to alter septin localization and organization. Given the increasing recognition of septins in promoting actin assembly and stabilizing actin bundles (62, 70, 71, 72), it is possible that RID-mediated *N*^ε^-fatty-acylation of septin proteins and disruption of septin organization may also contribute to the collapse of the actin cytoskeleton. Our work thus provides an additional clue to understand RID-mediated cytoskeletal disruption. It is also worth mentioning that the whole septin interacting complex may be extensively affected by RID. We show that ANLN, an adaptor protein that recruits soluble septins to actin bundles (62), and CDC42EP4, a Cdc42 effector protein associated with septin hetero-oligomers and actin stress fibers (82), are within the septin interaction network (Fig. 2*E*) and both *N*^ε^-fatty-acylated by RID (Fig. 1*F*), although the functional consequences of these modifications remain to be characterized. Therefore, RID appears to exploit its fatty-acyltransferase activity to target multiple host proteins for broadly compromising various components of cytoskeletons, including actin, septins, and adaptors, either synergistically or independently in different host cell types.

Septins are known to play key roles not only in cytoskeletal organization (72), but also in host defense against bacterial infection (54). For example, septins are recruited to the actin tails of intracytosolic bacteria (*e.g.*, *Listeria monocytogenes* and *S. flexneri*) and assemble into ring-like structures around the actin tails and bacteria (83, 84). In the case of *S. flexneri* infection, septins further form cage-like structures to entrap actin-polymerizing bacteria that are restricted from replication and targeted to antibacterial autophagy (84, 85). By contrast, although septins are present at the site of entry for *L. monocytogenes*, the bacteria are not compartmentalized by septin cages due to the counteraction of the secreted ActA effector (86). In the case of *Pseudomonas aeruginosa* infection, septins accumulate at the site of bacterial attachment and form a rigid barrier below the membrane to prevent bacterial internalization into host cells (87). During *Vibrio* infection, while disruption of the actin cytoskeleton by RID of the MARTX toxin is well-characterized, relatively little is known about the involvement of septins in comparison to actin. Our present data demonstrate the direct covalent modification of septins mediated by RID and suggest an interplay between septins and *Vibrio* species as well as a potential role of septins in host defense against these pathogens. In the efforts to compare the substrates of RID and IcsB, we show that IcsB can also directly modify septin proteins including SEPT6, SEPT7, and SEPT9 (supplemental Fig. S8*A*). Similarly, septin filaments were disrupted in IcsB-WT-expressing cells (supplemental Fig. S8, *B* and *C*), and septin proteins were more resistant to TX100 compared to those in IcsB-C306A-expressing cells (supplemental Fig. S8, *D* and *E*). These results thus suggest that virulent effectors of different bacterial origins utilize a similar mechanism involving *N*^ε^-fatty-acylation to manipulate host cell signaling. Notably, IcsB was previously shown to suppress septin caging of *Shigella* in HeLa cells (84). It would be interesting to examine in the future whether IcsB-mediated *N*^ε^-fatty-acylation of septins contributes to the inhibition of septin cage assembly during *Shigella* infection, especially considering that the modification limits the relative abundance of cytoplasmic septin pool.

In summary, the chemical proteomics profiling of RID-mediated *N*^ε^-fatty-acylation described herein has not only uncovered many new protein targets of RID of the MARTX toxin but also revealed the functional consequences of RID-mediated *N*^ε^-fatty-acylation on manipulating septin localization, organization, and dynamics. While our current understanding of RID function is largely limited to inducing host cell rounding, the discovery of new RID targets in host cells should greatly facilitate the functional analysis of this critical effector domain during *Vibrio* infection. With the characterization of RID-mediated *N*^ε^-fatty-acylation of septins, our study highlights the crucial roles of *N*^ε^-fatty-acylation and septins in host–pathogen interactions and expands the scope of PTMs in regulating septin assembly. Lastly, given that the study of *N*^ε^-fatty-acylation in mammalian cells has significantly lagged behind that of other types of long-chain fatty-acylation, our study also provides important insights into the function and regulation of this understudied type of protein fatty-acylation.

## Data Availability

The mass spectrometry proteomics data for the chemical proteomic profiling and AP-MS experiments have been deposited to the ProteomeXchange Consortium (http://www.proteomexchange.org/) *via* the PRIDE (88) partner repository with the dataset identifiers PXD046500 and PXD046497, respectively. The annotated spectra for chemical proteomic profiling and AP-MS experiments have been deposited to MS-Viewer with search keys guvlfwwvno and abkbai0nbr, respectively.

## Supplemental data

This article contains supplemental data (31).

## Conflict of interest

The authors declare that they have no known competing financial interests or personal relationships that could have appeared to influence the work reported in this paper.
