# Supplementary material for: Chemical Proteomics Reveals Nε-Fatty-Acylation of Septins by Rho Inactivation Domain (RID) of the Vibrio MARTX Toxin to Alter Septin Localization and Organization
Source: Mol Cell Proteomics. 2024 Feb 2;23(3):100730. doi: 10.1016/j.mcpro.2024.100730 (PMC10924143; doi:10.1016/j.mcpro.2024.100730)
Supplement: Supplemental Figures S1–S8 [file mmc5.pdf]

Supplemental Data for

Chemical proteomics reveals N^{ε}-fatty-acylation of septins by Rho Inactivation Domain (RID) of the *Vibrio* MARTX toxin to alter septin localization and organization

Yaxin Xu[1], Ke Ding[1], and Tao Peng[1,2]*

[1] State Key Laboratory of Chemical Oncogenomics, School of Chemical Biology and Biotechnology, Peking University Shenzhen Graduate School, Shenzhen 518055, China
[2] Institute of Chemical Biology, Shenzhen Bay Laboratory, Shenzhen 518132, China

Supplemental Figures S1–8.
Supplemental Table S1. Quantitative chemical proteomic analysis of the N^{ε}-fatty-acylation substrates of RID.
Supplemental Table S2. Bioinformatics analysis of RID substrates identified in the quantitative chemical proteomics.
Supplemental Table S3. Comparison of RID and IcsB substrates.
Supplemental Table S4. Affinity-purification and mass spectrometry analysis of SEPT6 interacting proteins.

Supplemental Figures

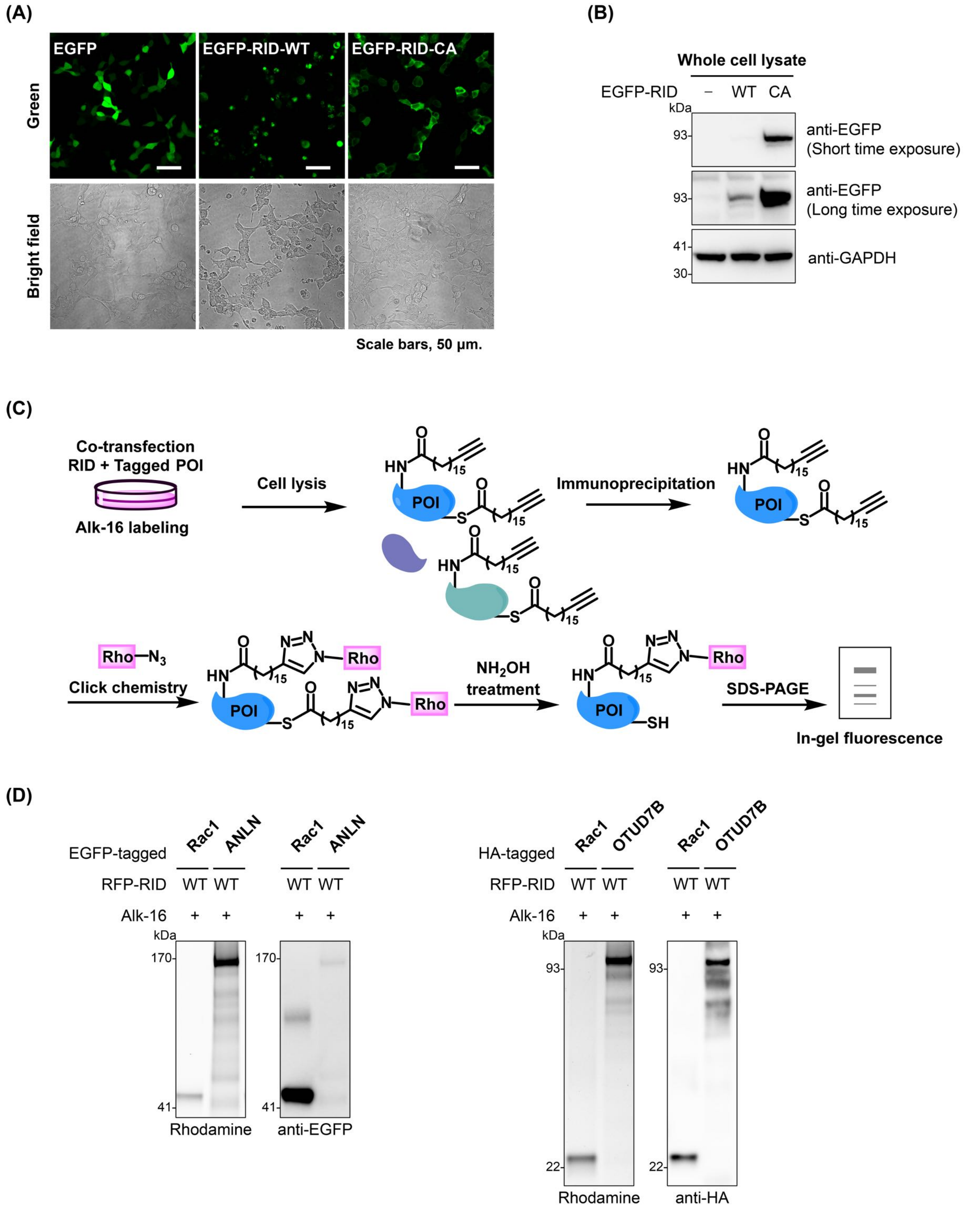

Figures S1. Expression of RID in mammalian cells and analysis of RID-mediated N^{ε}-fatty-acylation of candidate proteins. (A) Fluorescence imaging to analyze the expression of EGFP-tagged RID-WT and its inactive mutant RID-CA. HEK293T cells were transfected with EGFP-tagged RID-WT and RID-CA plasmids and analyzed by fluorescence microscopy. (B) Western blotting to analyze the expression of EGFP-tagged RID-WT and its inactive

mutant RID-CA. HEK293T cells were transfected with EGFP-tagged RID-WT and RID-CA plasmids and analyzed by Western blotting. (C) Workflow for analysis of RID-mediated N^{ε}-fatty-acylation of candidate proteins of interest (POI). HEK293T cells were co-transfected with RID and individual tagged POI, metabolically labeled with Alk-16, and lysed. The cell lysates were subjected to immunoprecipitation, click reaction with azido-rhodamine, NH_2OH treatment to cleave thioester bonds, and in-gel fluorescence analysis. (D) Validation of RID-mediated N^{ε}-fatty-acylation of RAC1 and other candidate proteins (e.g., ANLN and OTUD7B) identified in the quantitative chemical proteomics. Samples were prepared as in (C). Anti-HA or anti-EGFP immunoblotting is shown to confirm sample loading.

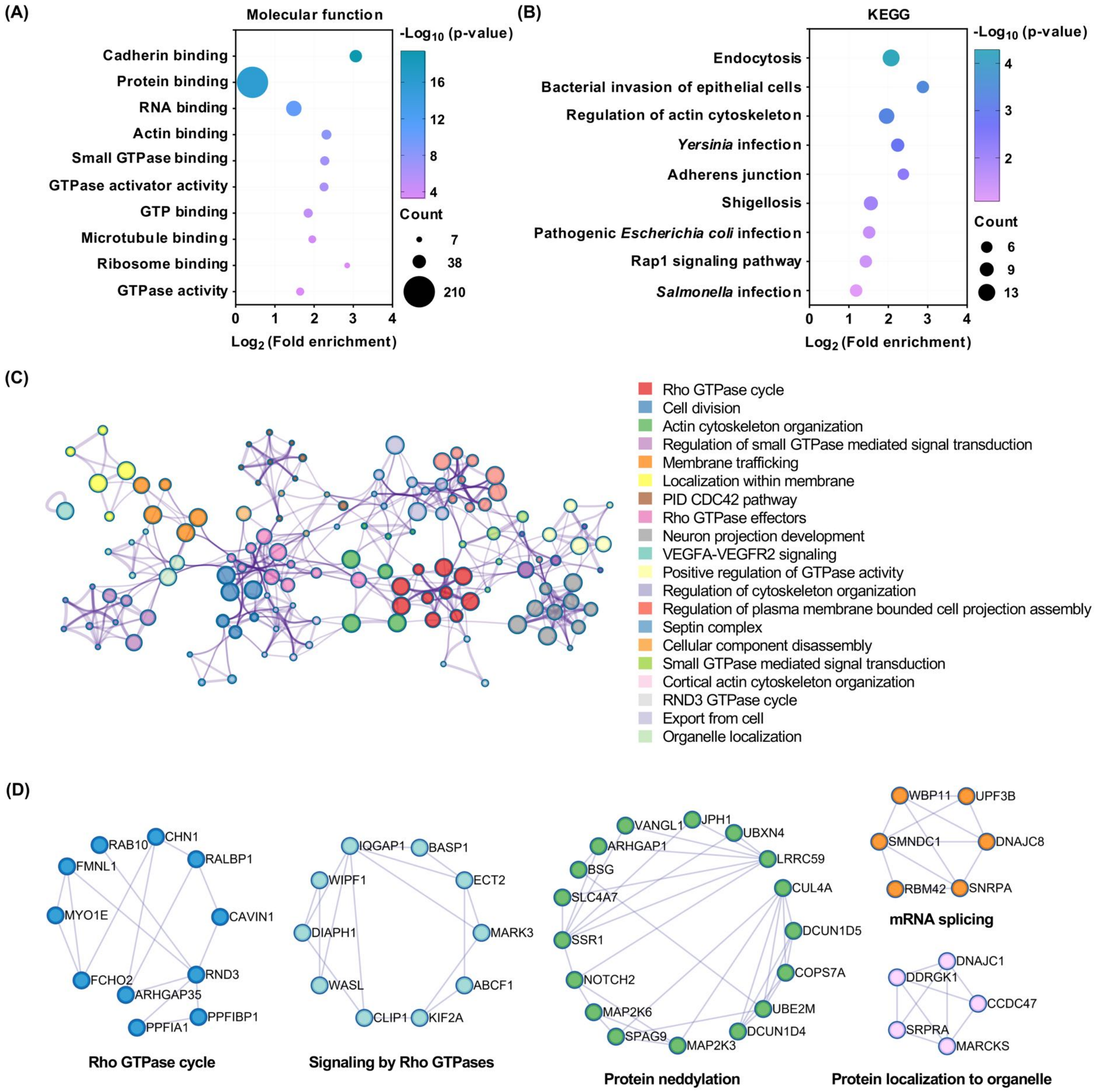

Figure S2. Bioinformatics analysis of the candidate RID substrates. (A) GO molecular function enrichment analysis of the candidate RID substrates. (B) KEGG pathway enrichment analysis of the candidate RID substrates. (C) Metascape enrichment network visualization showing the intra-cluster and inter-cluster similarities of enriched terms. Each node represents an enriched term and nodes are connected by similarities. Cluster annotations are shown in color code. (D) Protein complexes identified in the network of the candidate RID substrates by Metascape. Significantly enriched ontology terms are combined to annotate the putative biological roles of these protein complexes.

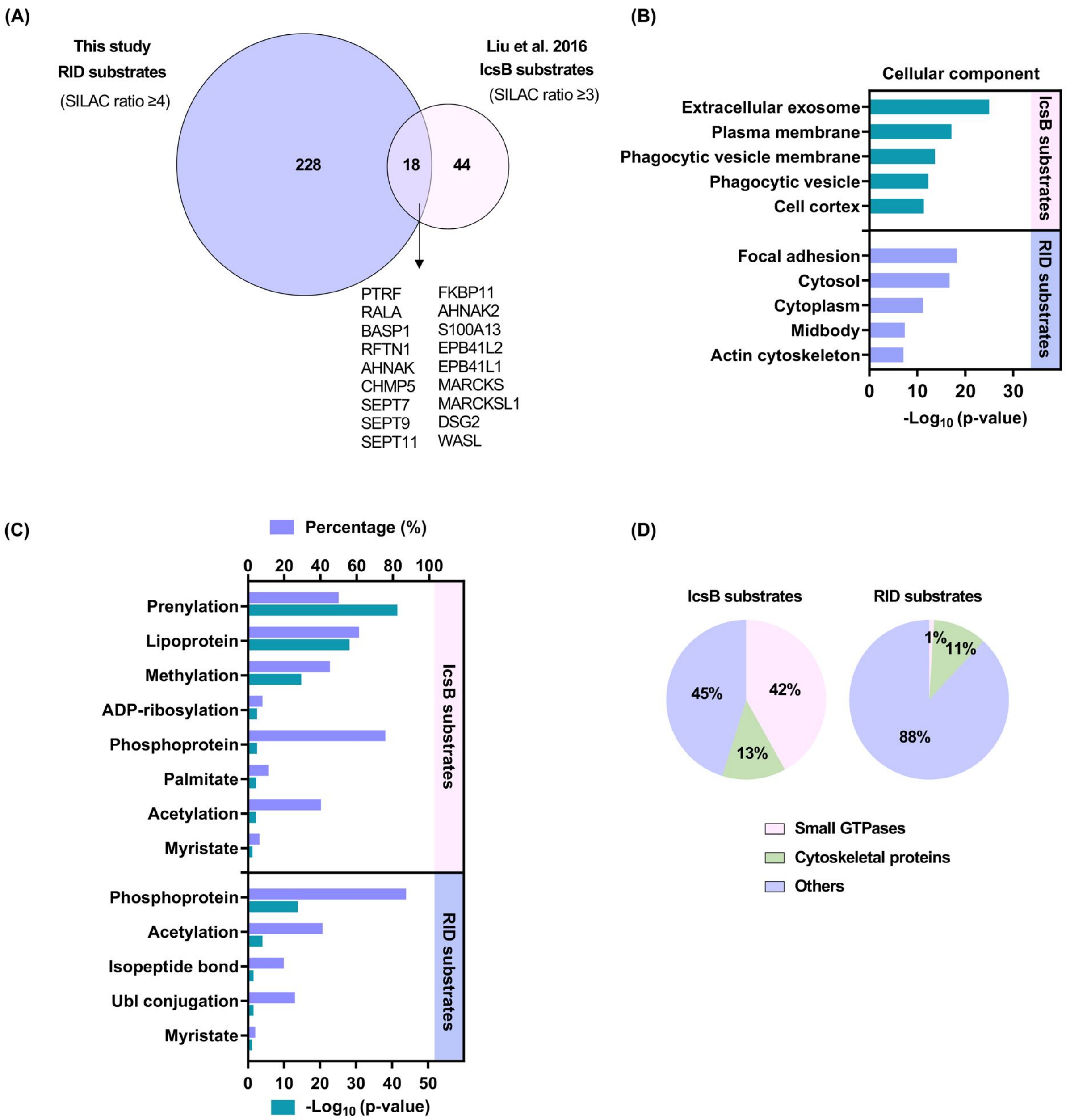

Figure S3. Comparison of candidate RID substrates and IcsB substrates. (A) Cross-reference of candidate RID substrates and IcsB substrates. IcsB substrates were identified in a previous study (*Nat Microbiol* 2018, 3, 996-1009). (B) Comparison of the cellular component enrichment analyses of RID substrates and IcsB substrates. (C) Comparison of the PTM enrichment analyses of RID substrates and IcsB substrates. (D) Comparison of the protein class analyses of RID substrates and IcsB substrates.

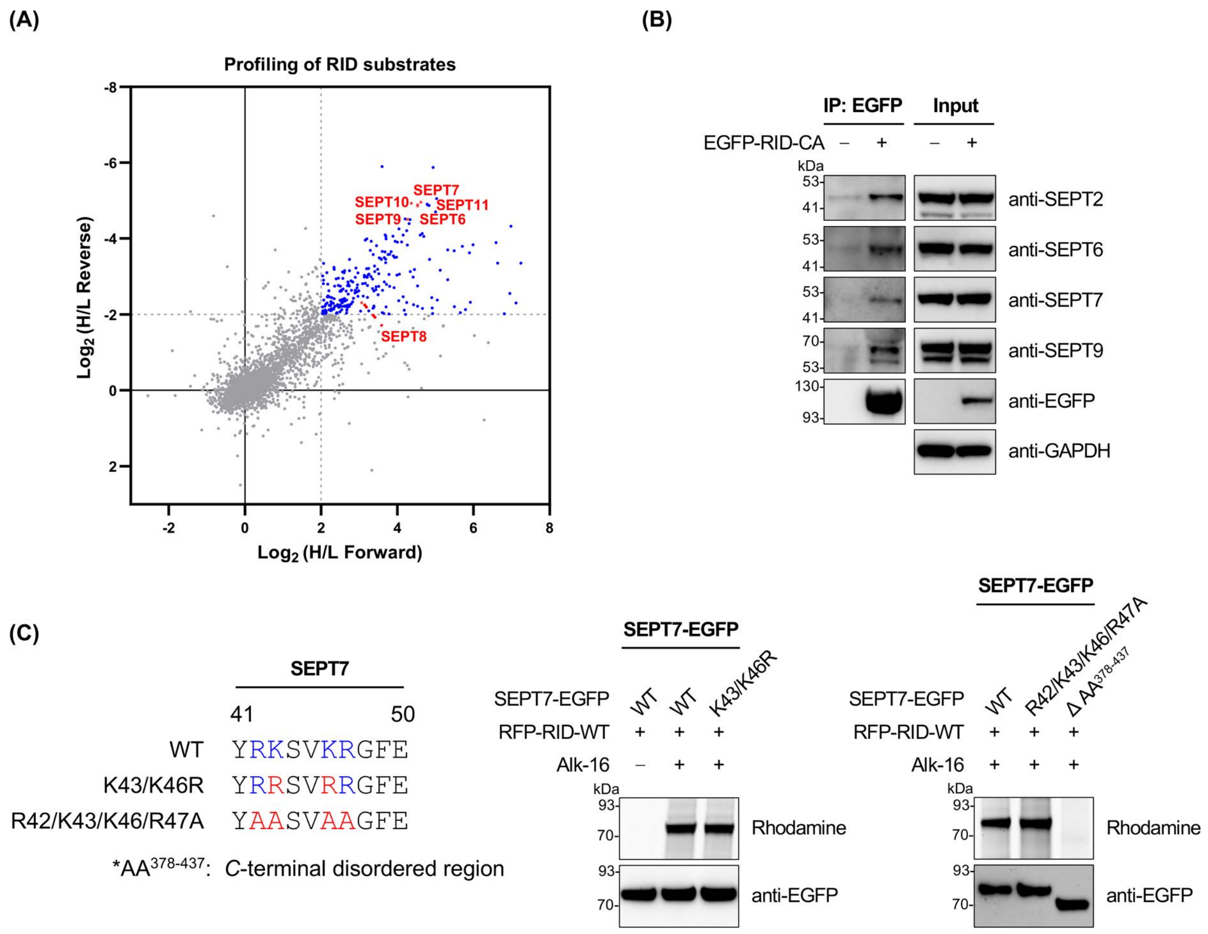

Figure S4. Analysis of RID-mediated *N*^ε^-fatty-acylation of septin proteins. (A) Scatter plot of the SILAC quantitative chemical proteomics data. H/L represents the SILAC ratio between heavy and light labels in the indicated Forward or Reverse experiment. Shown in red are representative septin proteins. (B) Co-immunoprecipitation analysis of RID-CA with septin proteins. HEK293T cells were transfected with EGFP-tagged RID-CA and subjected to co-immunoprecipitation and Western blotting analysis. (C) Effects of lysine mutation in the *N*-terminal PBR of SEPT7 on RID-mediated *N*^ε^-fatty-acylation.

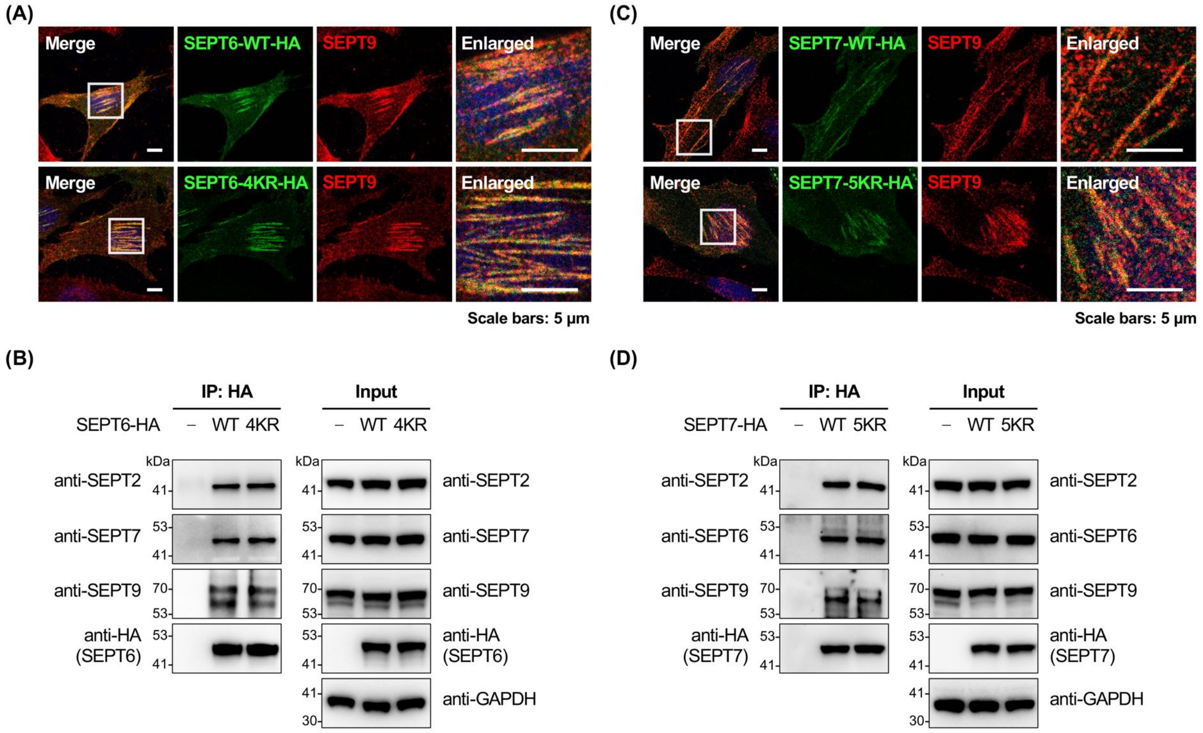

Figure S5. Colocalization and co-immunoprecipitation analysis of HA-tagged SEPT6 and SEPT7 with endogenous septins. (A) Immunofluorescence imaging of HA-tagged SEPT6 and endogenous SEPT9. HeLa cells were transfected with HA-tagged SEPT6 or its lysine-to-arginine mutant SEPT6-4KR and processed for anti-HA and anti-SEPT9 immunostaining. The enlarged images show the magnified areas in the white rectangles. (B) Validation of interactions between HA-tagged SEPT6 and endogenous septins by co-immunoprecipitation and Western blotting. HEK293T cells were transfected with HA-tagged SEPT6 or its lysine-to -arginine mutant SEPT6-4KR, using the empty vector as the control, processed for anti-HA immunoprecipitation, and analyzed by Western blotting. (C) Immunofluorescence imaging of HA-tagged SEPT7 and endogenous SEPT9. HeLa cells were transfected with HA-tagged SEPT7 or its lysine-to-arginine mutant SEPT7-5KR and processed for anti-HA and anti-SEPT9 immunostaining. The enlarged images show the magnified areas in the white rectangles. (D) Validation of interactions between HA-tagged SEPT7 and endogenous septins by co-immunoprecipitation and Western blotting. HEK293T cells were transfected with HA-tagged SEPT7 or its lysine-to -arginine mutant SEPT7-5KR, using the empty vector as the control, processed for anti-HA immunoprecipitation, and analyzed by Western blotting.

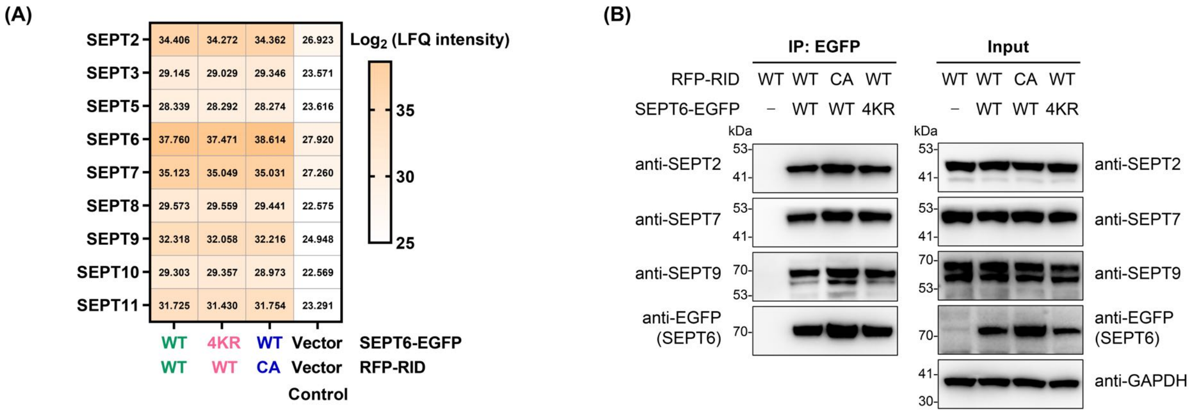

Figure S6. Affinity-purification and mass spectrometry analysis of SEPT6 interacting proteins. (A) LFQ intensities of septin proteins identified and quantified as SEPT6 interacting proteins in the affinity purification and mass spectrometry analysis. HEK293T cells were co-transfected with the indicated plasmids, using corresponding vectors without RID and SEPT6 inserts as the control, and processed in three biological replicates for affinity purification, in-gel digestion, and proteomic identification and quantification. (B) Validation of septin proteins as SEPT6 interacting proteins by co-immunoprecipitation and Western blotting. HEK293T cells were co-transfected with the indicated RID and SEPT6 plasmids, processed for anti-EGFP co-immunoprecipitation, and analyzed by Western blotting.

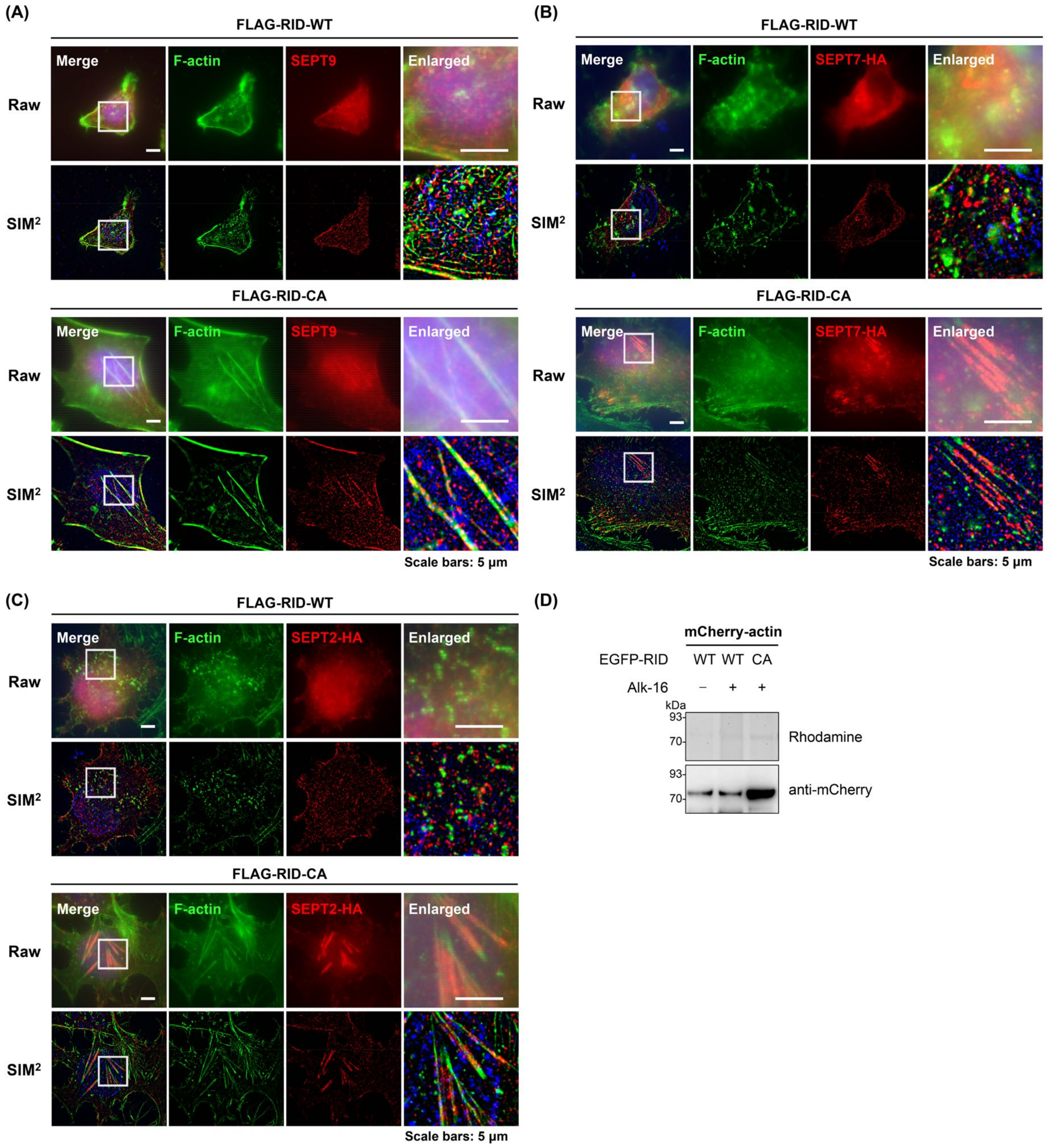

Figure S7. Analysis of the actin cytoskeleton in the presence and absence of RID activity. (A–C) Fluorescence imaging of the actin cytoskeleton in the presence and absence of RID activity. HeLa cells were transfected with RID or its inactive mutant and processed for (A) anti-SEPT9 immunostaining and F-actin staining with phalloidin. HeLa cells were co-transfected with HA-tagged (B) SEPT7 or (C) SEPT2 and RID or its inactive mutant and processed for anti-HA immunostaining and F-actin staining with phalloidin. (D) In-gel fluorescence analysis of RID-mediated N^{ε}-fatty-acylation of actin. HEK293T cells were co-transfected with actin and RID or its active mutant, metabolically labeled with Alk-16, and subjected to in-gel fluorescence assay.

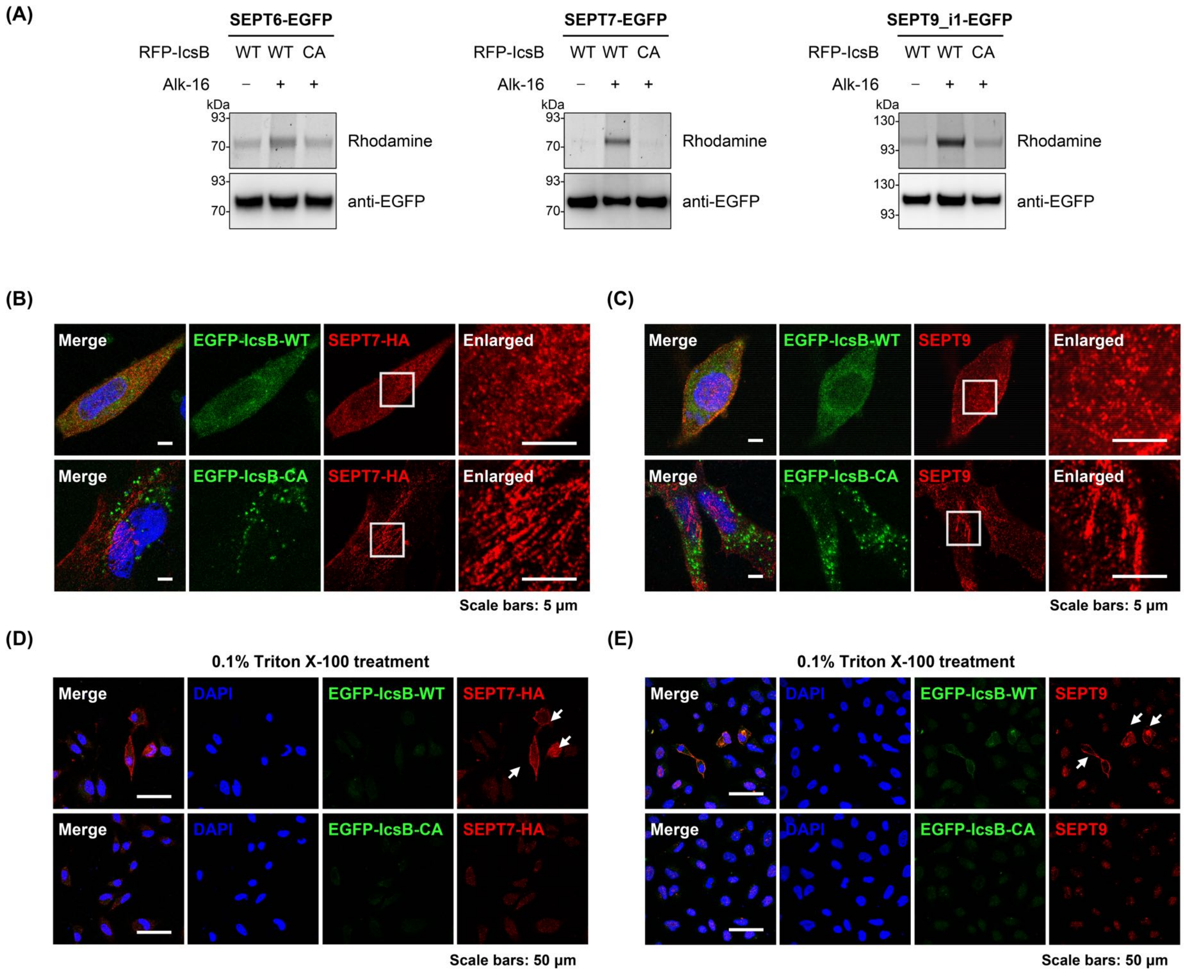

Figure S8. IcsB-mediated N^{ε}-fatty-acylation of septin proteins. (A) In-gel fluorescence analysis of IcsB-mediated N^{ε}-fatty-acylation of septin proteins. HEK293T cells were co-transfected with individual septin proteins (e.g., SEPT6, SEPT7, and SEPT9) and IcsB or its active C306A mutant (IcsB-CA), metabolically labeled with Alk-16, and subjected to in-gel fluorescence assay. (B–C) Immunofluorescence imaging of the organization and assembly of septin proteins, e.g., (B) SEPT7 and (C) SEPT9, without TX100 pretreatment in the absence and presence of IcsB activity. (D–E) Immunofluorescence imaging of the organization and assembly of septin proteins, e.g., (D) SEPT7 and (E) SEPT9, after TX100 pretreatment in the absence and presence of IcsB activity.
